# The placental maturation inflection point: an integrative biological framework for understanding early-onset versus late-onset preeclampsia

**DOI:** 10.3389/fendo.2026.1899767

**Published:** 2026-09-01

**Authors:** Ho Yen Chueh, Chia Lin Chang

**Affiliations:** 1 Department of Obstetrics and Gynecology, Chang Gung Memorial Hospital, Linkou Medical Center, Taoyuan, Taiwan; 2 Chang Gung University College of Medicine, Taoyuan, Taiwan

**Keywords:** early-onset, human placental lactogen, late-onset, placental maturation, placental senescence, preeclampsia, sFlt-1/PlGF ratio, vasculosyncytial membrane

## Abstract

**Background:**

Preeclampsia (PE) is clinically classified into early-onset (EOPE, <34 weeks) and late-onset (LOPE, ≥34 weeks) phenotypes that differ in epidemiology, angiogenic profiles, and outcomes. Although the 34-week cutoff is widely adopted, its biological rationale remains formally unarticulated in terms of placental developmental biology.

**Objective:**

To propose and evaluate the Placental Maturation Inflection Point (PMIP) — the transition from parenchymal expansion (Phase I) to functional optimization (Phase II) at approximately 34–36 weeks — as an integrative biological framework explaining the EOPE/LOPE phenotype division. The PMIP is conceptualized as a distributed developmental transition zone (~30–36 weeks) rather than a discrete biological event at a single gestational age.

**Methods:**

Narrative synthesis integrating evidence from placental morphometry, villous stereology, molecular trophoblast biology, endocrine and angiogenic biomarkers, imaging, and senescence biology, with structured engagement of competing frameworks.

**Results:**

Seven converging, partly interrelated lines of evidence may identify a placental developmental transition zone spanning ~34–36 weeks: (1) volumetric growth deceleration on MRI; (2) vasculosyncytial membrane attenuation and terminal villus predominance by stereology; (3) molecular regulators of syncytialization (p45 NF-E2, GCM1, Syncytin-1) showing trajectory shifts consistent with maturation; (4) human placental lactogen (hPL) near-plateau at ~34 weeks, reflecting maximal syncytiotrophoblast mass; (5) placental growth factor (PlGF) peak at ~30 weeks and subsequent soluble fms-like tyrosine kinase-1 (sFlt-1)/PlGF ratio shift; (6) placental calcification accelerating after the 36-week Grannum threshold; and (7) differential senescence and stress-pathway activation in EOPE versus LOPE. Evidence strength varies across pillars and is graded explicitly. No prior framework has anchored the 34-week cutoff to a convergent multi-domain placental developmental transition.

**Conclusions:**

The PMIP offers a biologically plausible framework for understanding why the 34-week cutoff has proven clinically productive. Within this hypothesis, EOPE reflects a pattern consistent with arrested Phase I, in which defective placentation may prevent functional maturity, whereas many LOPE cases may arise in the setting of a relatively mature placenta whose capacity is exceeded by maternal demand. Mixed phenotypes are expected; EOPE evidence is multi-stranded while LOPE evidence rests primarily on one molecular comparison and absence-of-pathology inference. This framework has implications for biomarker interpretation, therapeutic windows, and trial design.

## 1 Introduction

Preeclampsia complicates 2–8% of pregnancies worldwide and remains a leading cause of maternal and perinatal morbidity and mortality, responsible for an estimated 50,000 maternal deaths and 500,000 perinatal deaths annually (1, 2). The clinical heterogeneity of preeclampsia has long been recognized, but a major conceptual advance occurred when von Dadelszen, Magee, and Roberts proposed subdividing the syndrome into early-onset preeclampsia (EOPE, presenting before 34 weeks of gestation) and late-onset preeclampsia (LOPE, presenting at or after 34 weeks) in 2003 (3). This dichotomy was based on the observation that EOPE and LOPE differ not only in timing but in their epidemiological risk factors, hemodynamic profiles, angiogenic biomarker signatures, placental pathology, and maternal and neonatal outcomes (4–8).

**Figure 1:**
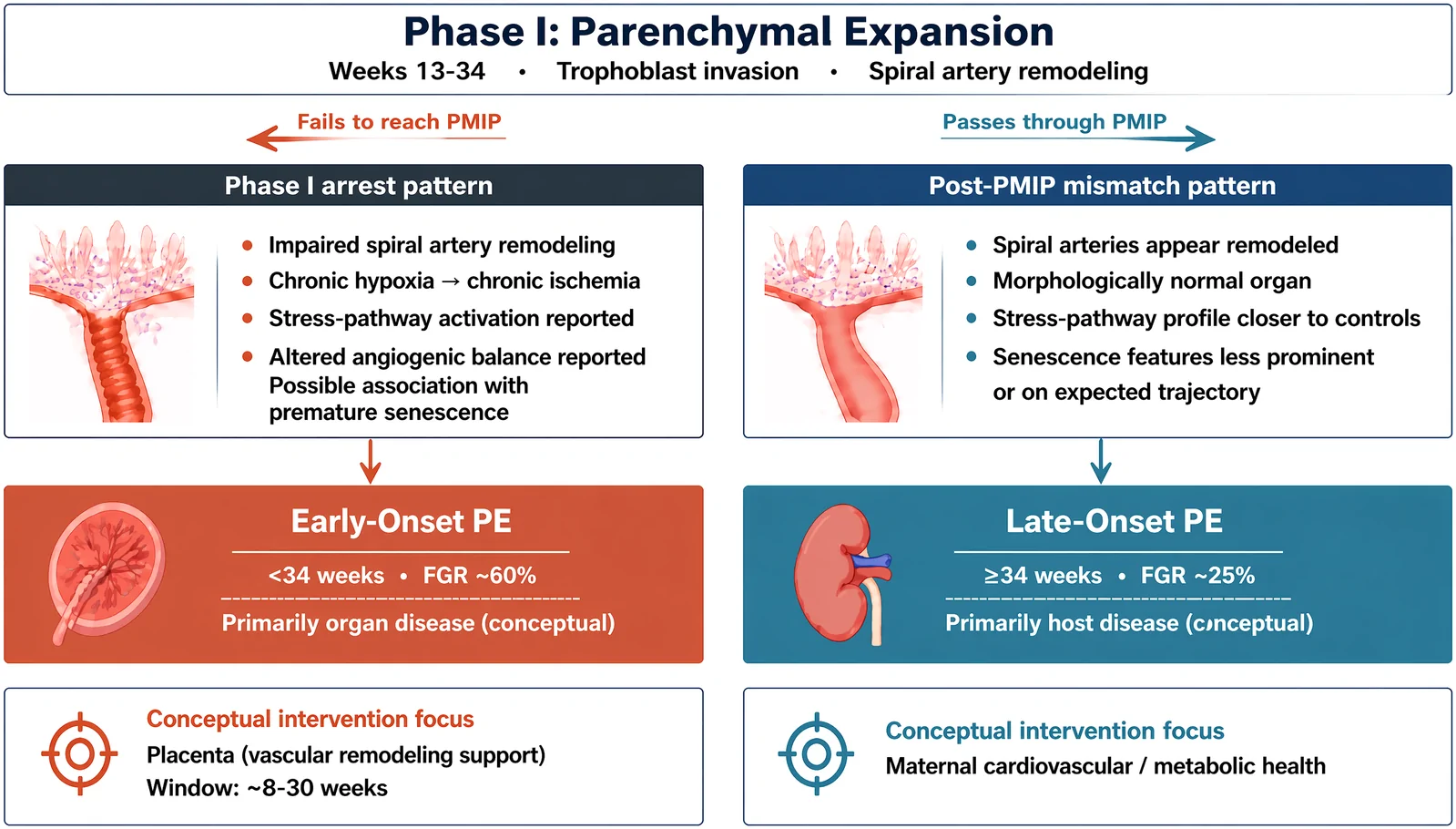
Proposed PMIP framework: predominant phenotypic patterns in EOPE versus LOPE. This schematic illustrates the conceptual PMIP framework for preeclampsia phenotyping. Starting from Phase I parenchymal expansion (weeks 13–34), the PMIP (~34–36 weeks) depicts the proposed developmental boundary. Left pathway (EOPE): the placenta is proposed to fail Phase I completion due to impaired spiral artery remodeling, resulting in a pattern consistent with arrested Phase I, including chronic intervillous hypoxia, ischemia, stress-pathway activation reported (62), pathological anti-angiogenic shift (sFlt-1/PlGF ratio ≥85) (50, 51), and features consistent with premature senescence (P16 expression 7.51× controls, 52.6% short telomeres in one study) (61). Right pathway (LOPE): the placenta is proposed to traverse the PMIP and achieve Phase II functional maturation, with a stress-pathway profile reported to be closer to normotensive controls in one study (62) and senescence proceeding on its normal schedule (60, 61). Disease is proposed to arise from mismatch between the mature placenta’s fixed functional capacity and maternal cardiovascular or metabolic demand.

The distinction has proven clinically productive. EOPE, affecting ~10–20% of preeclampsia cases, is characterized by defective spiral artery remodeling, severe angiogenic imbalance (markedly elevated sFlt-1 and suppressed PlGF; reviewed in (97)), high rates of concurrent fetal growth restriction (FGR), and worse maternal and neonatal outcomes (4, 5). The placental pathology of EOPE is dominated by vascular malperfusion, including villous infarction, decidual vasculopathy, and accelerated villous maturation (9). Conversely, LOPE, comprising ~80–95% of cases, is increasingly recognized as a disorder of maternal cardiovascular and metabolic adaptation. LOPE is characterized by a largely normal placenta, lower FGR rates (~25%), less severe angiogenic imbalance, and a more favorable prognosis (5, 8, 10) (**Figure 1**).

The 34-week cutoff is applied as an empirical convention, established primarily through clinical outcome stratification and epidemiological risk factor differentiation. A large population-based cohort study from La Réunion Island (n≈1,736 preeclamptic deliveries) systematically tested gestational-age cutoffs from 30 to 37 weeks and validated that 34 weeks best discriminates maternal risk profiles: chronic hypertension showed an adjusted odds ratio (aOR) of 6.0–6.5 for EOPE versus lower values for LOPE; history of prior preeclampsia showed an aOR of 12–17 for EOPE; body mass index (BMI) associated more strongly with LOPE at all cutoffs; and gestational diabetes was protective against EOPE (aOR 0.42–0.73 from cutoff 30–34 weeks) but showed no association with LOPE (10). Yet the paradigm has never been anchored in placental biology. The result is an unresolved puzzle: the 34-week cutoff has proven productive in clinical practice, trial design, and biomarker interpretation, yet it has never been explained why this particular gestational age — and not 30 or 38 weeks — delineates phenotypes that may reflect fundamentally different etiologies. Recent reviews have recognized the pathophysiological divergence between EOPE and LOPE, attributing EOPE to abnormal placentation and LOPE to maternal cardiovascular and metabolic predisposition (5). Huppertz has argued that existing preeclampsia theories “cannot explain the whole spectrum of symptoms and timing” and called for new hypotheses grounded in trophoblast biology (11). The PMIP framework is consistent with early trophoblast pathology as the initiating event. However, no prior work has identified the specific placental developmental transition that explains why the clinical phenotype diverges at 34 weeks rather than at another gestational age.

**Figure 2:**
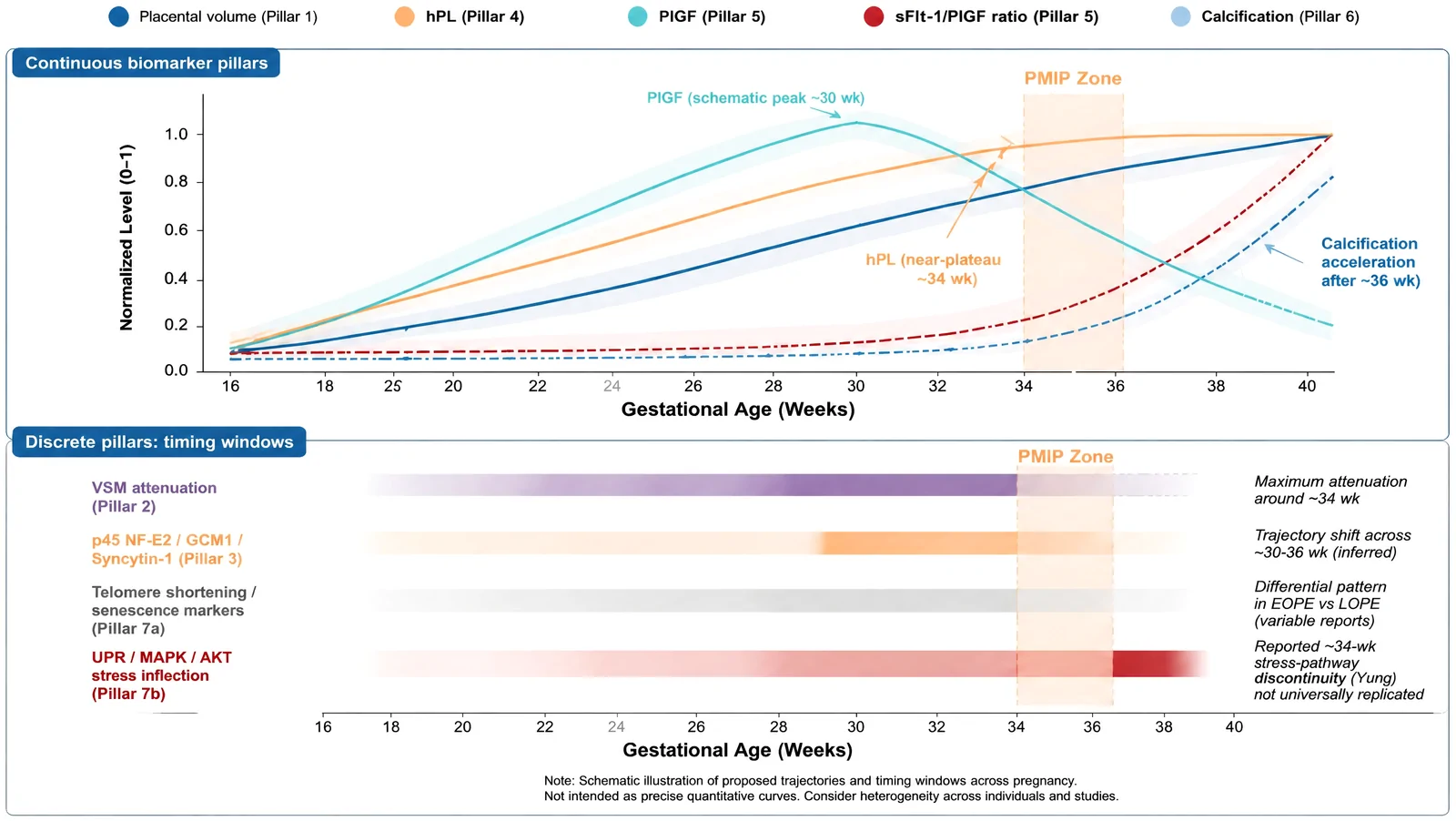
Schematic integrative timeline of seven proposed evidence pillars across gestation (varying evidence strength; see **Table 1**). All seven candidate pillars supporting the PMIP hypothesis are represented on a schematic gestational age axis (weeks 12–40) with their characteristic timing windows shown as shaded bars or trajectory curves. Pillar 1: placental volumetric growth deceleration on MRI (18–20). Pillar 2: vasculosyncytial membrane attenuation and terminal villus predominance from stereology (26–31). Pillar 3: trajectory shift from p45 NF-E2/Syncytin-1 expression (Phase I) to GCM1 activity (Phase II) (39–44). Pillar 4: hPL concentration rising to near-plateau at 34 weeks (45, 46). Pillar 5: PlGF peaking at ~30 weeks then declining, with sFlt-1/PlGF ratio inflecting upward (48–51). Pillar 6: placental calcification index acceleration after the 36-week Grannum threshold (57–59). Pillar 7a: differential telomere and senescence dynamics in EOPE versus LOPE (60, 61). Pillar 7b: 34-week molecular stress pathway inflection (Yung et al.) (62). The shaded PMIP zone (34–36 weeks) marks the central convergence window of a distributed transition spanning ~30–36 weeks. Note: curves and bars are schematic approximations, not precise quantitative summaries; evidence strength varies substantially across pillars.

Meanwhile, a rich and largely separate literature on placental developmental biology has established that the human placenta undergoes a fundamental transition in the mid-to-late third trimester: a shift from exponential parenchymal expansion (which is termed Phase I) to functional optimization through vasculosyncytial membrane (VSM; the thin trophoblast layer separating fetal capillaries from maternal blood) attenuation and transport efficiency enhancement (Phase II) (12–14). This transition is governed by specific molecular regulators of trophoblast differentiation, accompanied by convergent endocrine, angiogenic, and structural changes that cluster in the 34–36 week gestational window (**Figure 2**).

To our knowledge, no prior work has systematically integrated these two literatures to show that the EOPE/LOPE division reflects a fundamental inflection point in placental developmental biology. This narrative review proposes the Placental Maturation Inflection Point (PMIP) as an integrative biological framework, assembles converging evidence from seven converging domains (**Table 1**), and evaluates both supporting and contradictory data. We define the PMIP not as a fixed time point but as a distributed developmental transition zone—typically spanning 34–36 weeks—during which the placenta shifts from growth-dominant (Phase I) to efficiency-dominant (Phase II) function. We present the PMIP as a testable hypothesis, not as established consensus. The term “pre-PMIP” refers to the Phase I growth period before this transition, and “post-PMIP” refers to the Phase II optimization period after it. Within this framework, EOPE reflects a predominantly organ-driven disease in which the placenta is arrested in Phase I and does not reach functional maturity—while LOPE reflects a predominantly host-driven disease in which the placenta has largely completed maturation and its functional capacity is exceeded by maternal demand. These are dominant phenotypic tendencies rather than absolute biological classes; overlap and mixed presentations are expected and are addressed in Sections 9.3, 9.6, and 9.7.

**Table 1:** Seven converging evidence pillars supporting the placental maturation inflection point (PMIP) framework.

Pillar	Evidence domain	Key finding and trends	Timing	Refs	Strength of evidence
1	Placental volumetry (MRI)	Cubic polynomial growth curve; 4/20 women plateau at 34–38 wk; T2* declines linearly — volumetric deceleration with structural consolidation.	Plateau: ~34–38 wk	(18–20)	Weak (volumetric plateau in 4/20 women only)
2	Villous stereology & VSM attenuation	Terminal villi ~40% at term; VSM diffusion distance ~3.7 μm; villous surface area >12 m². Progressive transport optimization via membrane thinning.	Complete: ~36 wk	(16, 17, 26, 28–30)	Moderate
3	Molecular syncytialization regulators	p45 NF-E2 declines →GCM1 derepressed → Syncytin-1 decreases; HERV genes ↓ in IUGR. Shift from active fusion to functional optimization.	3rd trimester shift	(39–44)	Weak (no human longitudinal data across 34–36 wk; trajectories inferred)
4	hPL trajectory	hPL near-plateau at ~34 wk, reflecting active STB mass. Near-plateau parallels structural maturation; strong candidate circulating PMIP biomarker.	Near-plateau: 34 wk	(45–47)	Strong
5	PlGF & sFlt-1/PlGF ratio	PlGF peaks ~30 wk then declines; sFlt-1/PlGF ratio shifts with GA-specific cutoffs. Angiogenic program completion drives ratio rise.	PlGF peak: ~30 wk; ratio shift: ~34 wk	(48–52)	Moderate (PlGF peak at ~30 wk, not 34 wk)
6	Placental calcification (Grannum grading)	Grade III <36 wk = pathological (SGA, FGR); Grade III ≥37 wk = physiological. 36-wk threshold parallels PMIP.	Pathological/physiological threshold: 36 wk	(57–59)	Weak (Grannum inter-rater reliability and clinical utility contested)
7a	Telomere dynamics & senescence markers	TL decreases with GA (not PE-specific); EOPE shows premature senescence (↑P16, ↑SA-β-Gal, short TL) vs near-normal LOPE. LOPE aging is reactive (oxidative, SOD-rescuable (53)); EOPE aging is constitutive (developmental arrest, not rescuable) — supporting EOPE-as-organ-disease vs LOPE-as-host-disease distinction.	GA-dependent; premature in EOPE	(53, 60, 61)	Moderate (modest sample sizes; n=26 and n=224 cohorts)
7b	Molecular stress pathways	UPR, MAPK, HSP70 ↑ in <34 wk PE; ≥34 wk PE indistinguishable from controls; AKT ↓ only <34 wk.	Molecular inflection: 34 wk	(62)	Strong but unreplicated

Evidence strength assessment (author-rated based on study design, sample size, and temporal specificity): Strong — Pillars 4 (hPL near-plateau) and 7b (molecular stress inflection). Moderate — Pillars 2 (VSM attenuation/villous stereology), 5 (PlGF; sFlt-1/PlGF ratio), and 7a (differential placental senescence markers; modest cohort sizes, n=26 and n=224). Weak — Pillars 1 (placental volumetric plateau), 3 (molecular regulators p45 NF-E2, GCM1, Syncytin-1), and 6 (Grannum inter-rater reliability and clinical utility contested). Pillar 7b, which is independent of any clinical threshold-setting, is the strongest molecular anchor for the framework.

This review is organized into four phases. First, Sections 3–7 present the biological foundation: we present the biphasic model of placental growth (Section 3) and assemble seven lines of evidence — drawn from histology and molecular biology (Section 4), endocrinology and angiogenic biomarkers (Section 5), macroscopic imaging (Section 6), and senescence biology (Section 7) — that converge on the 34–36 week transition zone. Second, Section 8 develops the integrative model in which EOPE is proposed to represent organ disease (arrested Phase I) and LOPE is proposed to represent host disease (post-PMIP metabolic mismatch). Third, Sections 9 and 10 critically evaluate the framework, addressing nine potential counter-arguments and outlining the future research that would falsify or refine the hypothesis. Finally, Sections 11–13 translate the framework into a multi-modality assay panel for PMIP-stratified diagnosis, discuss implications for therapeutic-window optimization and trial design, and conclude.

## 2 Methods

### 2.1 Search strategy and databases

We performed a narrative literature search in PubMed/MEDLINE, supplemented by Scopus and Google Scholar for completeness, between September 2025 and March 2026. Reference lists of retrieved reviews were examined for additional relevant publications. The search was not registered, no PRISMA flow was generated, and no formal risk-of-bias assessment was applied. This is consistent with the hypothesis-generating intent of the synthesis.

### 2.2 Search terms

Searches combined domain terms (placental maturation, vasculosyncytial membrane, villous stereology, GCM1, p45 NF-E2, syncytin-1, hPL, PlGF, sFlt-1, placental calcification, placental senescence) with disease terms (preeclampsia, early-onset preeclampsia, late-onset preeclampsia, fetal growth restriction) using Boolean operators.

### 2.3 Inclusion and exclusion criteria

Sources prioritized for inclusion were: quantitative morphometric, stereological, or volumetric studies of human placental development across gestation; molecular data on trophoblast differentiation regulators (GCM1, p45 NF-E2, Syncytin-1) and their gestational trajectories or dysregulation in pregnancy complications; longitudinal or cross-sectional data on circulating biomarkers (hPL, PlGF, sFlt-1) across the third trimester; histopathological studies of delayed villous maturation in the context of preeclampsia or FGR; molecular studies comparing EOPE and LOPE placentas; imaging studies of placental calcification or functional parameters; and epidemiological or clinical studies validating the EOPE/LOPE classification. Both original research and reviews were considered. Non-human studies were consulted only where translational relevance was direct.

### 2.4 Data extraction and synthesis

From each source we noted study design, sample size, gestational age range, and key quantitative findings relevant to the seven evidence domains described in Sections 3–7. Convergence of partly related evidence streams at the 34–36 week gestational window was evaluated qualitatively. This review is hypothesis-generating, not a formal systematic review or meta-analysis. Each pillar is graded for evidence strength (strong/moderate/weak) in **Table 1**, distinguishing direct empirical evidence from inferred trajectories or single-study findings.

### 2.5 Roadmap

The biological convergence at this transition is supported by seven converging domains of evidence — acknowledging that several share mechanistic links and are not statistically independent (see Section 8 for discussion of inter-pillar relationships) — which constitute the core scaffolding of this review: (1) placental volumetric trajectories by MRI (Pillar 1, Section 3.2); (2) vasculosyncytial membrane attenuation and villous stereology (Pillar 2, Section 4.1); (3) molecular regulators of syncytialization — p45 NF-E2, GCM1, and Syncytin-1 (Pillar 3, Section 4.4); (4) human placental lactogen near-plateau (Pillar 4, Section 5.1); (5) placental growth factor peak and the sFlt-1/PlGF ratio shift (Pillar 5, Section 5.2); (6) placental calcification and the 36-week Grannum threshold (Pillar 6, Section 6.1); and (7) placental senescence markers and the 34-week molecular stress inflection (Pillars 7a and 7b, Section 7). The temporal convergence of these Pillars is summarized in **Table 1** and illustrated in **Figures 1** and **2**; a condensed quick-reference format is provided in **Box 1**.

Box 1 **The seven pillars at a glance.**

Pillar 1 (Volumetric growth deceleration; MRI; ~34–38 wk; weak — plateau in 4/20 women only). Pillar 2 (Vasculosyncytial membrane attenuation, terminal villus predominance; stereology; ~34 wk; moderate). Pillar 3 (p45 NF-E2/GCM1/Syncytin-1 trajectory shift; molecular biology; ~34 wk; weak — inferred trajectories, no human longitudinal data). Pillar 4 (hPL near-plateau; circulating biomarkers; ~34 wk; strong). Pillar 5 (PlGF peak with sFlt-1/PlGF ratio shift; circulating biomarkers; ~30 wk; moderate). Pillar 6 (Placental calcification acceleration; ultrasound; ~36 wk; weak — Grannum reliability contested). Pillar 7a (Differential telomere/senescence dynamics; senescence biology; GA-dependent, premature in EOPE; moderate — n=26 and n=224 cohorts). Pillar 7b (UPR/MAPK/AKT stress inflection in EOPE; molecular biology; 34 wk; strong but unreplicated). Format: (evidence domain; primary timing window; strength of evidence).

## 3 The biphasic model of placental growth

### 3.1 Phase I: parenchymal expansion (weeks 13–34)

After structural organogenesis is complete by ~12 weeks (12, 14), the placenta enters an extended period of exponential tissue growth that constitutes Phase I. During this period, the chorionic villi undergo extensive branching angiogenesis (the formation of new vessels by sprouting from existing capillaries to expand the vascular tree), developing increasingly dense and complex capillary networks to accommodate the exponentially rising metabolic demands of the developing fetus (12, 15). Stereological studies applied to cross-sectional samples of human placentas collected at 10–41 weeks of gestation showed that expansion of total villous volume and surface area is driven by dramatic linear growth of terminal villi beginning at approximately the middle of the second trimester (16). This linear growth is accompanied by progressive villous maturation involving increases in the relative volume of fetal capillaries and villous capillarization, coupled with decreases in villous diameter and thickness of the villous membrane (16).

Fetoplacental angiogenesis during Phase I is itself biphasic in character: an initial phase of capillary branching predominates through mid-gestation, followed by a transition to non-branching angiogenesis in the late second and early third trimesters (15). Branching angiogenesis generates new vessel segments that increase surface area but also increase flow resistance, whereas non-branching angiogenesis (capillary loop elongation) reduces resistance and promotes flow (15). Allometric analyses provide a quantitative framework for Phase I growth dynamics. Mayhew showed that villous growth matches the maternal intervillous space and overall placenta but falls behind that of the fetus (17), and villous volume is outpaced by surface area expansion and longitudinal villus growth. Within villi, fetal capillary volume grows faster than trophoblast, which grows faster than stroma — reflecting progressive production of mature terminal and intermediate villi with small diameters, thinner trophoblast, and reduced stroma (17). Placental growth is not isometric but gradually remodeled toward structures optimized for exchange. The switch, occurring at ~25 weeks, initiates a gradual transition from vessel creation to vessel optimization—that continues over the subsequent 9–11 weeks and culminates in the functional maturation plateau at 34–36 weeks.

### 3.2 Evidence Pillar 1: Placental volumetric growth transition (Phase II: weeks 34–36 to term)

*In vivo* volumetric data are consistent with — though do not unambiguously demonstrate — a transition in late third-trimester placental growth. A longitudinal MRI study of 20 normal primiparous women scanned seven times from weeks 14 to 38 found that mean placental volume increased linearly with a constant mean growth rate of ~30 mL/week, and the median volume extrapolated to delivery was 856 mL (range 602–1,050 mL) (18). Notably, only 4 of these 20 women (20%) showed placental volumes that plateaued between the final two scans (approximately weeks 34–38), consistent with the proposed maturation plateau (18). A larger cross-sectional MRI study (n=112) fitted placental volumetric data to a third-order polynomial curve in gestational age (GA), described as Volume = −0.02×GA³ + 1.6×GA² − 13.3×GA + 8.3. While placental volume showed positive correlations with both estimated fetal weight (p=0.03) and birth weight (p=0.05), it showed nonlinear growth with inflection in the mid-third trimester (19).

Complementary data from placental transverse relaxation time (T2*) relaxometry—a surrogate marker for tissue oxygenation and structural integrity—provide additional insight. In a mixed cohort of 85 pregnancies, placental T2* showed a significant negative linear correlation with gestational age (coefficient = −3.9 per week), while placental volume showed a significant positive linear correlation (20). This linear coefficient summarizes average volumetric growth across the full gestational range studied and is not inconsistent with the PMIP framework: the higher-order cubic polynomial fit cited above (19) resolves the same trajectory into early acceleration followed by deceleration in the late third trimester as the placenta enters Phase II. This divergence—increasing volume alongside decreasing T2*—reflects the transition from tissue generation to structural consolidation, with increasing tissue density, syncytial knotting, and calcification accompanying maturation. Notably, placental T2* was the only independent predictor of small-for-gestational-age (SGA) outcome in multivariate analysis (OR 0.81, p=0.04) (20), underscoring the functional significance of this maturation process.

A recent (January 2026) automated MRI volumetric pipeline (357 healthy term-control scans, 16–41 weeks) confirmed that placental volumes increase across gestation while amniotic fluid volume peaks mid-pregnancy and declines toward term (21), supporting differential growth trajectories of uterine compartments in the third trimester. The APPLAUSE pipeline (Automatic Prediction of PLAcental health via U-net Segmentation) further demonstrated that machine learning–derived placental maturation Z-scores from T2* maps correlate with histopathological maternal vascular malperfusion and accelerated aging, providing proof-of-concept for real-time placental maturation assessment (22).

### 3.3 The demand–supply paradox

**Figure 3:**
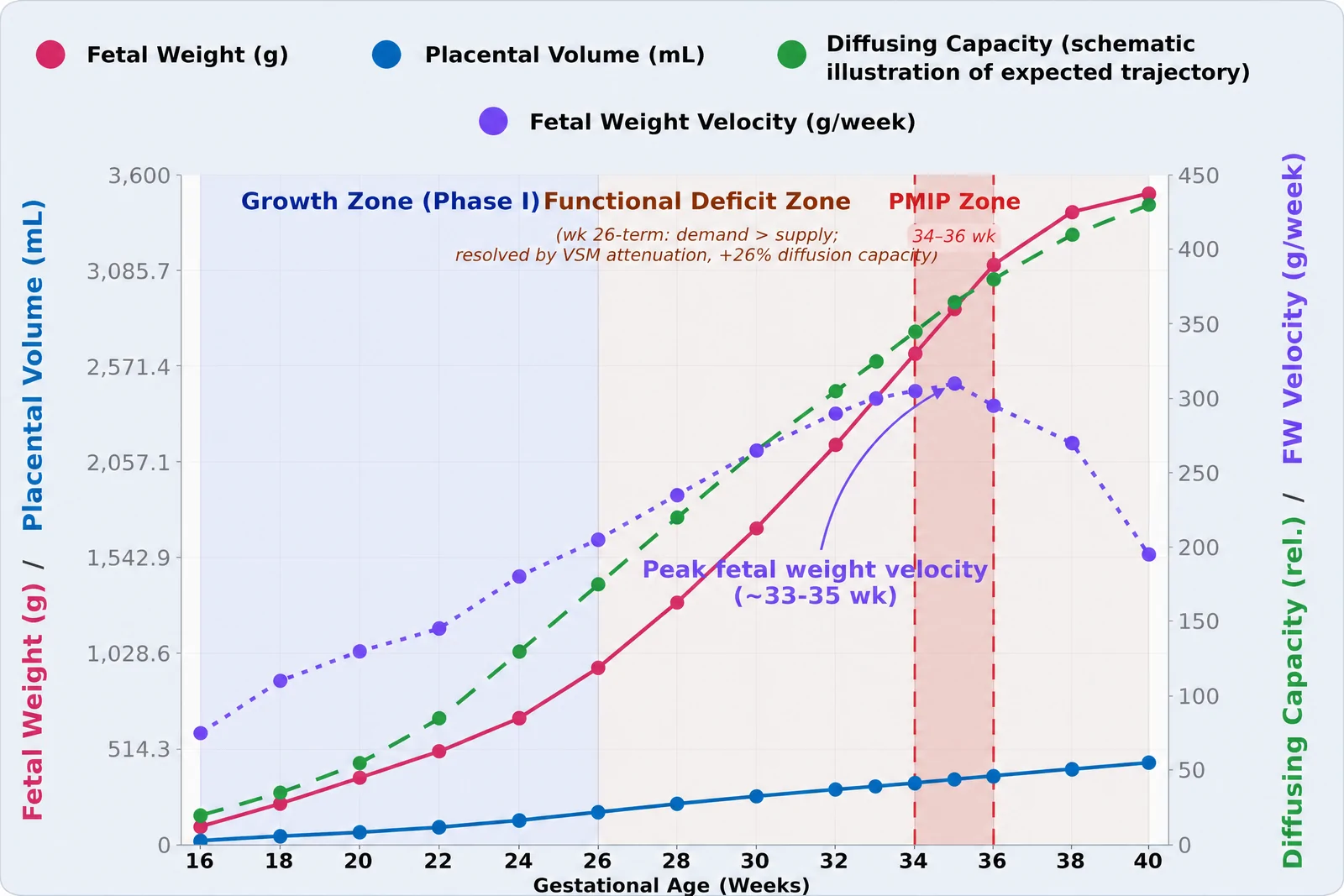
Conceptual schematic of the late-pregnancy demand–supply relationship and proposed Phase II adaptive optimization. The dual Y-axis plots shows that the growing demand-supply mismatch between placental volume (blue, placental capacity; decelerating) and fetal weight [red, fetal demand; exponential, with up to 7-fold increase across the second half of pregnancy (23)] during gestation is proposed to create increasing tension that may be partly accommodated by the maturation of placental diffusing capacity (green dashed, continuing to rise) before and after PMIP. VSM attenuation has been estimated to improve diffusing capacity by ~26% in morphometric modeling (28, 30). While the diffusing capacity continues to increase, the fetal weight velocity peaks at 33–35 weeks (24) (purple dashed line). The PMIP zone (red shade, 34–36 weeks) marks the transition from growth-driven to efficiency-driven placental function. Dual Y-axis scales note: fetal weight (g) and placental volume (mL) share the left axis (0–3,600) to make the magnitude of the demand–supply divergence visually apparent (fetal weight ~3,400 g at term vs. placental volume ~500 mL); fetal weight velocity is plotted on the right axis; literature peak values at 33–35 weeks range from ~220 g/week [NICHD standard references (24)] to ~300 g/week in some populations and growth charts, and the figure curve is drawn within this range as an illustrative trajectory. The axis maximum (450 g/week) provides headroom above the plotted peak. The diffusing capacity curve is shown as a schematic illustration of expected gradual increase across late gestation [shape based on published morphometric estimates; not plotted to a quantitative scale (28, 30)].

The fetal-to-placental weight ratio changes dramatically across gestation, creating the pressure that is proposed to drive Phase II optimization. In the second half of pregnancy, fetal weight increases up to seven-fold while the placenta’s relative villous surface area falls twofold relative to fetal mass (17, 23) (**Figure 3**). The mature placenta must therefore support a peaking increase in fetal metabolic demand despite an absolute halt in the creation of new functional tissue. Fetal weight velocity itself peaks at approximately 33–35 weeks of gestation — precisely at the leading edge of the PMIP window — and declines thereafter as term approaches (24) (**Figure 3**). The coincidence between peak fetal-weight velocity and the onset of Phase II is biologically suggestive: the metabolic load at 33–35 weeks could contribute to the physiological trigger that forces vasculosyncytial membrane attenuation and the transport-efficiency gains of Phase II, even as placental volumetric growth decelerates. We emphasize this is a candidate trigger hypothesis that requires prospective longitudinal validation (see Section 10.2), not a tested causal mechanism.

Critically, placental transport efficiency—the ease of transfer across the placental membrane—continues to increase even after the placental growth velocity peaks at approximately 30–32 weeks (25) — distinct from fetal weight velocity, which peaks later at 33–35 weeks (**Figure 3**). This paradox—increasing transport efficiency despite volumetric plateau—is the defining feature of Phase II. To resolve this paradox, the placenta undergoes microscopic architectural transformations that exponentially increase the transport efficiency of its existing structures—the very transformations that characterize Phase II and are described in Section 4.

## 4 Histological and molecular correlates of maturation

### 4.1 Evidence pillar 2: vasculosyncytial membrane attenuation and villous stereology

**Figure 4:**
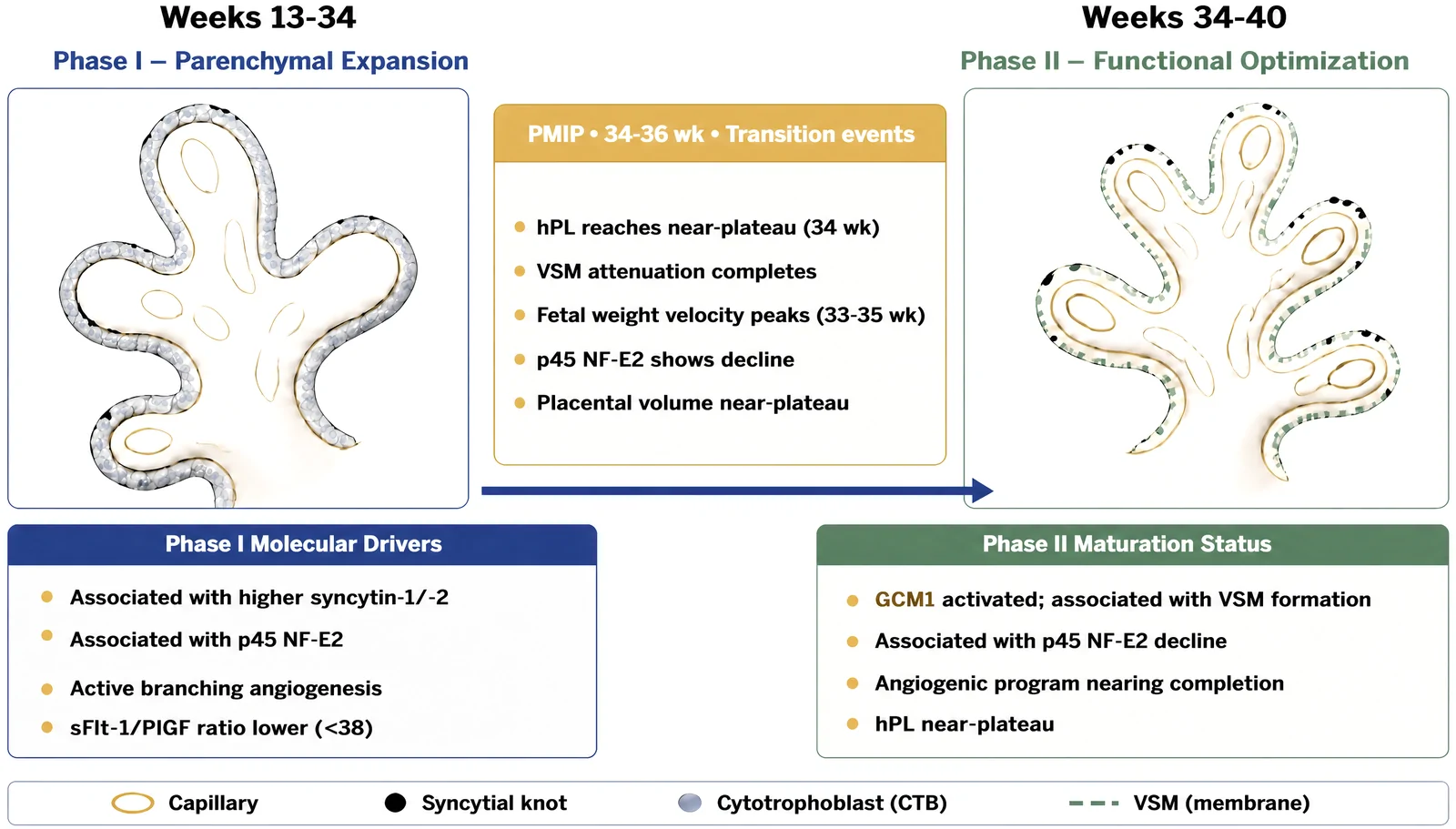
Proposed structural and molecular correlates of chorionic villous maturation across the Phase I→Phase II transition, illustrating conceptual links to Evidence Pillars 2 and 3. Left: Phase I villus (weeks 13–34) showing thick trophoblast, continuous cytotrophoblast, small central capillaries, and ~25 μm diffusion distance (27). Molecular associations: relatively higher Syncytin-1/2, higher p45 NF-E2, active branching angiogenesis, lower sFlt-1/PlGF ratio (42–44). Right: Phase II villus (weeks 34–40) showing dilated peripheral sinusoids forming VSMs (~3.7 μm) and syncytial knots. Villous surface area increases from ~1 m² to >12 m² (17, 26). Molecular associations: GCM1 activity associated with VSM formation, p45 NF-E2 declined, angiogenic program nearing completion, hPL near-plateau (39, 42, 45). Center: PMIP transition events (~34–36 weeks) — hPL near-plateau attainment, completion of VSM attenuation, peak fetal weight velocity [33–35 weeks; ref (24)], p45 NF-E2 derepression of GCM1, and placental volume deceleration.

The most important histological transformation defining peak placental maturity is formation of vasculosyncytial membranes (VSMs) — highly attenuated regions where fetal capillaries touch the trophoblast basement membrane, with syncytiotrophoblast nuclei and cytoplasm physically displaced to the villous periphery (12, 25). In the terminal villi of the mature placenta, the fetal capillary vessels and syncytiotrophoblast are separated by only a thin basement membrane, achieving a minimal maternal-fetal diffusion distance of approximately 3.7 μm (26) (**Figure 4**). This represents a dramatic reduction from the diffusion distances of ~25 μm characteristic of early and mid-gestational villi (27).

The functional consequence of VSM thinning can be quantified by stereological measurements of villous membrane thickness. Two complementary metrics are informative: arithmetic mean thickness (Ta), which weights all villous regions equally, and harmonic mean thickness (Th), which weights thinner regions more heavily because they contribute disproportionately to diffusion. At term, Ta ranges from 4.44 to 4.53 μm while Th ranges from 3.56 to 3.65 μm (28, 29). The resulting Ta/Th ratio of approximately 1.24 indicates that local membrane thinning at VSMs — together with the contribution of syncytial knots, focal aggregations of syncytiotrophoblast nuclei that sequester aging nuclear material away from active diffusion zones (Section 4.2) — reduces the effective diffusion resistance by approximately 26% compared with a hypothetical membrane of uniform thickness (28). This degree of functional gain becomes critical at term, when growing fetal demand approaches the limits of the placenta’s transport capacity.

The villous epithelium thins progressively during the third trimester because surface area expands faster than volume (29); microvillous surface area continues to increase until ~36 weeks (25). At term, terminal villi — grape-like structures with extensive capillarization, and minimal stroma — comprise nearly 40% of total villous volume and typically contain 4–6 fetal capillaries per cross-section, collectively providing a total villous surface area exceeding 12 m² — an extraordinary increase from just ~1 m² at mid-gestation (17, 26).

Progressive dominance of terminal villi, with VSM attenuation, could represent a structural endpoint of the Phase I→Phase II transition. Morphometric modeling shows that the most critical structural variable determining oxygen-diffusing capacity is the harmonic mean thickness of the villous membrane, followed by the surface areas of villi and fetal capillaries; on the other hand, blood space volumes and plasma diffusion distances (i.e., harmonic mean thickness of the maternal and fetal plasma layers separating erythrocytes from the villous membrane) have negligible effects by comparison (30). Thus, the VSM attenuation and surface-area amplification occurring during Phase II could be the primary mechanisms by which the placenta achieves its maximum transport capacity — and the maximum is reached at ~36 weeks.

### 4.2 Syncytial knots as maturation temporal markers

Syncytial knots (SKs) — focal aggregations of syncytiotrophoblast nuclei on the outer surface of tertiary villi — increase with gestational age and serve as quantitative markers of placental aging (12). Their formation reflects sequestration of aging nuclei away from metabolically active regions, preserving the efficiency of the surrounding trophoblast (12, 31). In normal pregnancies, SK prevalence rises gradually through the third trimester, and the diagnostic thresholds commonly applied in placental pathology are SKs in >33% of villi at term and >30% in preterm placentas (12). When they are beyond these levels, maternal vascular malperfusion is strongly suggested. Excessive SK formation, particularly at earlier gestational ages, is a common feature of EOPE, maternal vascular malperfusion, and FGR, and signals that the placenta has aged prematurely even though it has not completed Phase I (31). The SK burden is thus a crucial indicator to distinguish normal maturation from pathological hypermaturation, and it provides a conceptual bridge to the maturation disorders discussed in the next subsection.

### 4.3 Maturation disorders as disease mechanisms

When the placenta fails to transition from parenchymal expansion (Phase I) to VSM attenuation (Phase II) at the appropriate gestational time, delayed villous maturation (DVM) occurs. DVM is characterized by a monotonous villous population consisting of larger and fewer terminal villi with a lack of peripheral capillaries, low numbers of VSMs and syncytial knots for gestational age, increased villous stroma, and centralized capillarization resembling earlier gestational stages (32, 33). The standardized term “DVM” was proposed in the Amsterdam Placental Workshop Group Consensus Statement (32), and has been increasingly linked to late-term stillbirth and congenital heart disease (34, 35).

Conversely, accelerated villous maturation — preterm villi prematurely assuming term-like morphology (placental villous hypermaturation, PVH) — is a hallmark of maternal vascular malperfusion and is more common in preeclamptic pregnancies (31, 36). Importantly, a study using data from the National Collaborative Perinatal Project (2,525 preterm births, 24–34 weeks) found that PVH was associated with improved neonatal outcomes (survival, Apgar scores, oxygen use), supporting the hypothesis that the maturation program itself is not pathological but adaptive, and that accelerated maturation is a compensatory response to improve the transport capacity under stress conditions (36).

Japanese cohort data from the Hamamatsu Birth Cohort (HBC Study, n=258) independently confirmed that placental maturation disorders have lasting functional consequences. Both accelerated villous maturation and DVM were significant predictors of impaired infant development through the first 18 months of life (37), and of lower neurodevelopmental milestone scores through 40 months of age (38). These data provide independent validation that the maturation timeline must be precisely calibrated—both premature and delayed transitions produce harm.

### 4.4 Evidence pillar 3: molecular regulators of the maturation transition

Pillar 3 is built largely from inferred trajectories. No single longitudinal human study has quantified p45 NF-E2, GCM1 activity, and Syncytin-1 across the 34–36 week window. The supporting evidence is drawn from comparisons between normal and IUGR placentas combined with cross-sectional gestational-age datasets. The pillar is therefore best characterized as consistent with — rather than directly demonstrating — a 34–36 week molecular transition. The molecular regulators discussed below are general syncytialization regulators whose dysregulation in IUGR is mechanistically suggestive but not yet preeclampsia-specific.

The morphological shift from cytotrophoblast-rich proliferative villi to terminally differentiated syncytiotrophoblast-dominant structures with attenuated VSMs is regulated by a hierarchy of molecular regulators whose activity profiles align with the proposed PMIP. Glial cells missing-1 (GCM1) is a master transcription factor regulating syncytiotrophoblast differentiation in the human placenta. GCM1 controls the expression of the fusogenic genes Syncytin-1 and Syncytin-2, which mediate physical fusion of cytotrophoblast cells into the multinucleated syncytiotrophoblast (39, 40). GCM1 is also required for extravillous trophoblast (EVT) differentiation, the pathway responsible for spiral artery remodeling (41). Its expression level is reduced under hypoxic conditions in both first-trimester and term primary cytotrophoblasts, with consequent syncytia formation deficiency (39, 40). This directly links placental oxygenation status to the maturation program. GCM1 has been identified as among the top genes with the greatest negative association with fetal growth in intrauterine growth restriction (IUGR) placentas (40), and its reduction in mouse placenta leads to defective syncytiotrophoblast differentiation and gestational hypertension resembling preeclampsia (41).

The transcription factor p45 NF-E2 (nuclear factor erythroid derived 2) provides upstream regulatory control over GCM1. It negatively regulates syncytiotrophoblast differentiation by modulating GCM1 post-translational modifications (42). Expression of p45 NF-E2 is significantly reduced in human placentas complicated by IUGR compared with healthy controls. This reduction is associated with enhanced GCM1 acetylation and desumoylation, increased syncytiotrophoblast differentiation, and elevated apoptosis (42). In murine models, p45 NF-E2 deficiency causes IUGR and impaired placental vascularization independent of its role in megakaryopoiesis (43). p45 NF-E2 thus functions as a molecular brake on premature syncytialization: its hypothesized gradual downregulation during the third trimester may contribute to the Phase I→Phase II transition, while its pathological loss leads to excessive apoptosis and placental insufficiency.

Syncytin-1, an endogenous retroviral envelope protein of the human endogenous retrovirus W (HERV-W) family, is the direct effector of trophoblast cell-cell fusion. Its mRNA increases until the late first trimester then progressively decreases toward the late third trimester (44). This gestational trajectory is consistent with the Phase II transition: as the organ shifts from tissue generation to functional optimization, the demand for new fusion events diminishes. Importantly, all six HERV envelope genes studied—including both fusogenic (Syncytin-1, Syncytin-2) and non-fusogenic members—show downregulation in IUGR placentas compared to controls (44), indicating an impairment of the fusion machinery in growth-restricted pregnancies. These coordinated molecular trajectories—p45 NF-E2 declining, GCM1 activity maturing, and Syncytin-1 decreasing—are collectively consistent with the Phase I→Phase II transition and connect the structural evidence of Pillars 1–2 to the circulating biomarker evidence described in the next Section.

## 5 The endocrine signature of placental maturation

### 5.1 Evidence pillar 4: human placental lactogen trajectory

Among circulating placental biomarkers, human placental lactogen (hPL) provides the most direct readout of functional syncytiotrophoblast mass, the cell type whose maturation peaks at the PMIP. hPL, also known as human chorionic somatomammotropin (hCS), is a syncytiotrophoblast-secreted polypeptide hormone that reflects active placental mass and functional capacity (45). hPL shares structural homology with growth hormone and prolactin and can bind both receptors. In concert with placental growth hormone (hPGH), hPL modulates maternal metabolism to ensure an abundant glucose supply for transplacental transfer by inducing peripheral insulin resistance, reducing maternal glucose utilization, and increasing maternal lipolysis (45).

hPL increases gradually from detection at ~6 weeks post-conception to near-plateau levels at ~34 weeks, with limited additional rise through term (45, 46). A meta-analysis published in 2022 confirmed that hPL levels are positively related to placental mass and infant birthweight (47). In type 1 diabetes, hPL levels appear lower in early pregnancy (possibly reflecting delayed placental development) and higher in late pregnancy (possibly reflecting increased placental mass) (47)—a pattern consistent with hPL serving as a real-time indicator of syncytiotrophoblast functional capacity. The hPL near-plateau at 34 weeks parallels the proposed structural maturation plateau, making it a strong candidate circulating biomarker of the PMIP transition.

### 5.2 Evidence pillar 5: placental growth factor and the sFlt-1/PlGF ratio

Placental growth factor (PlGF) is an angiogenic factor expressed heavily by villous syncytiotrophoblasts. It promotes development, arborization, and maturation of the placental vascular system (48, 49). In healthy pregnancies, circulating maternal PlGF rises progressively during Phase I parenchymal expansion, mapping directly to the period of active branching angiogenesis, and peaks at ~30 weeks of gestation (48). After 30 weeks, as the placenta shifts from creating new vessels to optimizing existing ones, PlGF levels naturally decline—reflecting the gradual completion of the angiogenic program (48, 49). In pregnancies complicated by preeclampsia, PlGF is pathologically suppressed, and the degree of suppression correlates with disease severity and with the gestational age at onset. Earlier-onset cases show more severe PlGF suppression (49, 50).

Soluble fms-like tyrosine kinase-1 (sFlt-1) acts as a decoy receptor, binding PlGF and vascular endothelial growth factor (VEGF) to inhibit their angiogenic activity. During late pregnancy, especially as the placenta approaches senescence, sFlt-1 naturally increases while PlGF decreases (50). The sFlt-1/PlGF ratio has emerged as a clinically validated diagnostic and prognostic tool. A ratio of ≤38 has a negative predictive value of 98.6% for ruling out preeclampsia within one week (51). The National Institute for Health and Care Excellence (NICE) recommends PlGF-based testing between 20 and 34 + 6 weeks for suspected preeclampsia (52), and the same rule-out cutoff (≤38) applies to both gestational phases but with different sensitivity and specificity, while the rule-in cutoffs differ markedly (≥85 before 34 weeks versus ≥110 at or after 34 weeks) (50, 51).

The clinical utility of the sFlt-1/PlGF ratio may itself reflect dysregulation of the Phase I→Phase II transition. During Phase I, active branching angiogenesis requires high PlGF and low sFlt-1, producing a low ratio reflecting a healthy growing placenta. As Phase II begins and the angiogenic program completes, PlGF naturally declines and sFlt-1 rises—the ratio increases physiologically to reflect normal maturation. In EOPE, this shift occurs pathologically early, likely because the arrested Phase I angiogenic program fails to generate adequate PlGF while the ischemic syncytiotrophoblast overproduces sFlt-1. The resulting ratio elevation (often ≥85 before 34 weeks) likely reflects a placenta that never achieved angiogenic competence and maturation (50, 51). In LOPE, the ratio may also be elevated because the physiological Phase II decline in PlGF coincides with maternal factors that further stress the system; however, the pathological increment above the gestational-age-appropriate baseline is typically smaller than in EOPE. The higher numerical rule-in threshold for LOPE (≥110 after 34 weeks versus ≥85 before 34 weeks (50)) reflects the fact that the normal baseline sFlt-1/PlGF ratio rises in late pregnancy as PlGF declines physiologically, not greater disease severity. This framework explains why gestational-age-specific ratio cutoffs are clinically necessary: the underlying angiogenic biology differs fundamentally before and after the PMIP.

Within the PMIP framework, the sFlt-1/PlGF ratio carries different biological meaning before and after the transition. In arrested Phase I, the chronically hypoxic placenta upregulates sFlt-1 as a maladaptive response to failed vascular development — the organ produces anti-angiogenic signals precisely because its angiogenic program was never completed. By contrast, in the post-PMIP placenta, the angiogenic program has already completed physiologically, and the sFlt-1 elevation in LOPE is consistent with senescence-driven release from a structurally mature organ rather than developmental failure (50–53).

### 5.3 Other endocrine markers

Beyond Pillars 4 and 5, several additional endocrine trajectories are consistent with the PMIP convergence. The free α-subunit of human chorionic gonadotropin peaks at ~36 weeks (54), placing it within the PMIP window; 17α-hydroxyprogesterone shows a second gradual rise from approximately week 32, reflecting placental utilization of fetal adrenal steroid precursors (55). By contrast, estradiol continues to rise through term rather than peaking at 34–36 weeks (56), excluding it from the convergence pattern. These observations are presented as supplementary to the seven-pillar architecture rather than as additional pillars.

## 6 Macroscopic imaging correlates of maturation

### 6.1 Evidence pillar 6: placental calcification and Grannum grading

The sixth line of evidence addresses macroscopic imaging correlates. Placental calcification — progressive deposition of calcium phosphate minerals in the vascular tissue — is the most prominent macroscopic sign of placental aging and the maturation transition. The Grannum grading system classifies placental maturation on ultrasound from Grade 0 (homogeneous) through Grade III (extensive indentations with echo-spared areas) (57). Calcification begins to increase at approximately 29 weeks, with a relatively steep rise in the percentage of placentas showing Grade II or Grade III calcification at 35–36 weeks (57, 58). A Grade III placenta at term (≥37 weeks) is regarded as a physiological aging process without negative clinical significance (57, 58).

Placental calcification before the maturation transition carries a fundamentally different clinical significance. Premature placental calcification (PPC) — Grade III, or combined Grades II and III, before 36 weeks — is a specific indicator of placental insufficiency (59). A 2025 retrospective study (n=3,088) confirmed that PPC before 36 weeks is significantly associated with SGA neonates and fetal growth restriction (58). Earlier studies have reported elevated relative risks for preeclampsia (RR 5.27; 95% CI 2.24–12.40), suspected fetal hypoxia (RR 1.71), preterm delivery (RR 2.11; 95% CI 1.00–4.45), and low Apgar scores in pregnancies with PPC (59).

The 36-week mark thus acts as a critical biological dividing line that parallels the PMIP. The widespread calcification after this point may reflect achievement of peak maturity and is clinically benign. On the other hand, calcification before this point signifies a pathological failure of the organ to sustain its growth phase—analogous to the premature senescence observed in EOPE placenta (see Section 7). As such, this macroscopic imaging correlate independently supports the PMIP concept and may have clinical utility as a non-invasive maturation marker.

However, Pillar 6 evidence comes with two caveats. First, Grannum grading is contested in modern practice. ACOG and SMFM no longer endorse it because of substantial inter-rater variability and limited prognostic value beyond other ultrasound parameters. Second, although the biological observation that calcification accelerates after 36 weeks is robust, the clinical translation via Grannum scoring is comparatively weak. The argument advanced here, therefore, rests on the underlying biology rather than on Grannum grading per se.

## 7 Placental senescence and completed maturation

### 7.1 Evidence pillar 7a: telomere dynamics, gestational aging, and differential senescence in EOPE versus LOPE

Pillar 7 — placental senescence — provides molecular evidence that the gestational age boundary between EOPE and LOPE coincides with a developmental transition. The placenta undergoes programmed cellular senescence as part of its normal lifecycle, with rapid growth, establishment of vascular supply, high metabolic output, and then orderly aging at term (12). Telomere dynamics provide a molecular readout of this process. A study using a large multi-ethnic cohort (n=224 placentas) has shown that placental telomere length (TL) decreases consistently with gestational age (delivery GA: beta = −0.029 per week, p=0.047) but is not associated with severe preeclampsia per se after controlling for gestational age difference (p=0.20) (60). This finding supports the concept that telomere attrition is a marker of normal placental aging—a programmed process inherent to the organ’s lifecycle—rather than a disease-specific pathology. However, senescence markers are differentially expressed between EOPE and LOPE placentas in ways that strongly support the PMIP framework.

Farladansky-Gershnabel et al. examined placental biopsies from 7 EOPE pregnancies, 6 LOPE pregnancies, and 13 gestational age-matched controls using quantitative FISH and senescence marker analysis (61). The percentage of trophoblasts with short telomeres was significantly higher in EOPE (52.61 ± 12.27%) versus LOPE (28.72 ± 10.14%), both of which were elevated relative to controls (7.53 ± 5.14%, p=0.03) (61). Telomere aggregate formation—a marker of dysfunctional telomere maintenance—was enhanced in EOPE (8.72 ± 2.49%) compared to LOPE (4.54 ± 1.45%) and controls (2.72 ± 1.08%, p=0.03) (61). Notably, P16 cDNA expression was approximately 7.5-fold higher in EOPE than in controls and 5.9-fold higher than in LOPE (n=7 EOPE, n=6 LOPE, n=13 controls; confidence intervals not reported), with senescence-associated beta-galactosidase (SA-β-Gal) staining significantly more positive in EOPE versus LOPE (61).

These findings indicate that EOPE may represent premature activation of the placental senescence program — accelerated aging of an organ that has not completed Phase I. The senescence is not a response to successful maturation (as in the normal term placenta) but a consequence of ischemic stress in a structurally deficient organ. In contrast, LOPE most likely occurs in an organ where senescence follows the normal temporal trajectory. The placenta has matured on schedule, and its programmed decline is proceeding normally.

Recent evidence further refines this picture: LOPE placentas exhibit accelerated molecular aging marked by telomere attrition and DNA damage, and oxidative stress — not inflammation — is proposed to drive this process (53). Antioxidant treatment with superoxide dismutase preserves telomere length and restores angiogenic balance. This result implies that reactive senescence can be rescued by antioxidants. This finding supports the PMIP framework’s characterization of LOPE as a host disease in which the Phase II placenta undergoes normal programmed senescence, but is reactively accelerated by extrinsic maternal oxidative burden, rather than the constitutive, premature senescence seen in EOPE’s arrested Phase I.

### 7.2 Evidence pillar 7b: the 34-week molecular stress inflection

Pillar 7b is the most direct molecular evidence for a 34-week inflection in the framework, but it is also the most methodologically vulnerable. It derives primarily from a single 2014 study by Yung and colleagues that, to our knowledge, has not been independently replicated by a separate research group (62). Despite this limitation, we include it as a pillar because of the biological specificity of its findings — but its strength will depend on prospective replication of these data.

The Yung et al. study — the single empirical foundation of Pillar 7b, which has not been independently replicated and which therefore requires confirmation in additional cohorts before its molecular discontinuity can be regarded as established — examined unfolded protein response (UPR; the endoplasmic reticulum stress response to misfolded proteins) pathways, mitogen-activated protein kinase (MAPK) stress pathways, heat-shock proteins, and AMP-activated protein kinase alpha (AMPKα) in placentas delivered by caesarean section at different gestational ages, with controls including second-trimester, preterm, and normal-term placentas (62). The study showed that activation of UPR markers including phosphorylated inositol-requiring enzyme 1 alpha (P-IRE1α), activating transcription factor 6 (ATF6), X-box binding protein 1 (XBP-1), glucose-regulated protein 78 (GRP78) and glucose-regulated protein 94 (GRP94), along with the MAPK stress marker P-p38/p38 and heat shock protein 70 (HSP70), was significantly higher in placentas from EOPE (<34 weeks) than in both LOPE (≥34 weeks) and normotensive controls, with a clear placental molecular inflection around 34 weeks (62).

Critically, LOPE (≥34 weeks) placentas and normotensive controls were molecularly indistinguishable: UPR and stress pathway activation profiles overlapped completely (62). AKT (protein kinase B), a central regulator of cell proliferation and survival during placental and fetal growth, was selectively reduced in <34-week PE placentas and in cells subjected to severe hypoxia-reoxygenation stress *in vitro*, but was not reduced in ≥34-week PE placentas or in any other condition (62). These data suggest that the molecular pathology of EOPE exists exclusively in the pre-PMIP window. Placental disease before 34 weeks produces a fundamentally different molecular signature from disease after 34 weeks, and disease after 34 weeks leaves the placenta molecularly intact.

## 8 Discussion

### 8.1 An integrative model of the PMIP

The PMIP framework does not compete with Redman’s two-stage model of preeclampsia (63) but provides its developmental biological mechanism. The two-stage model describes what happens — poor placentation (Stage 1) leading to maternal syndrome (Stage 2) — but does not explain why the clinical phenotype diverges so sharply at 34 weeks. The PMIP is proposed to identify the placental developmental transition that may determine whether PE arises from arrested parenchymal growth (EOPE) or from demand–supply mismatch in a structurally mature organ (LOPE). In this sense, the PMIP supplies the missing biological clock to the two-stage framework.

Plotting the seven pillars from Sections 3–7 on a single gestational age timeline reveals a transition zone spanning ~30–36 weeks, with a central tendency at 34–36 weeks (**Figure 2**; **Table 1**). Volumetric, structural, molecular, endocrine, angiogenic, calcification, and senescence pillars converge — at varying strengths of evidence and with several mechanistic interrelationships (e.g., PlGF dynamics and branching angiogenesis; hPL and syncytialization; calcification and stereological maturation) — within this window. The convergence is proposed to define the PMIP.

The PMIP is best understood as a transition zone, not a point. Different pillars inflect at slightly different gestational ages within this window — PlGF peaks at ~30 weeks, hPL near-plateau at ~34 weeks, calcification accelerates after 36 weeks — reflecting that placental maturation is coordinated but distributed rather than a switch. Individual PMIP timing variation plus cross-modality measurement noise is consistent with multi-pillar convergence. The 34-week clinical cutoff captures the central tendency of this distribution. It is neither fixed for every pregnancy nor arbitrary. A further caveat is that the seven pillars are not statistically independent. Vasculosyncytial membrane attenuation (Pillar 2) and Grannum calcification (Pillar 6) are both downstream consequences of completed terminal villus maturation, while the hPL near-plateau (Pillar 4) and PlGF decline (Pillar 5) both depend on the same syncytiotrophoblast functional state. The strength of the framework therefore lies in temporal convergence of mechanistically related observations rather than in convergence of fully independent measurements.

### 8.2 EOPE as organ disease: arrested Phase I

In this framework, EOPE is not proposed to represent premature activation of the PMIP. Rather, in the proposed PMIP model, EOPE is consistent with an arrested Phase I pattern: the organ may fail to complete its growth program because defective spiral artery remodeling in the first trimester may create a chronically hypoxic villous environment that prevents normal villous expansion and angiogenesis. The placenta is arrested before the PMIP, and EOPE could be best characterized as insufficient Phase I with reactive Phase II activation under ischemic stress as a compensatory response. However, premature acquisition of terminal-villus morphology cannot rescue the surface-area deficit from inadequate parenchymal expansion: EOPE placentas lack the parenchymal mass on which Phase II optimization depends. The molecular signatures observed in EOPE placentas — UPR activation, AKT suppression, sFlt-1 overproduction, and PlGF suppression — are consistent with chronic ischemia in a structurally incomplete organ, and are distinct from the patterns reported in LOPE or normotensive controls in the available comparative datasets (62). These observations fit the proposed framework, though causal relationships and generalizability require prospective confirmation. Accelerated villous maturation in EOPE may represent a compensatory attempt to increase transport capacity, but accelerated membrane thinning cannot compensate for the severely reduced total surface area from failed parenchymal expansion (36). Similarly, the premature senescence markers (elevated P16, SA-β-Gal, short telomeres) (61) might reflect ischemia-driven cellular damage, not age-appropriate programmed decline. Mechanistically, the bridge from arrested Phase I to clinical EOPE phenotype runs through chronic villous hypoxia activating HIF-1α, which transactivates the sFlt-1 gene. Concurrent endoplasmic reticulum (ER) stress activates the unfolded protein response, generating syncytiotrophoblast-derived microparticles and pro-inflammatory mediators that propagate maternal endothelial dysfunction. In EOPE, the placenta is both the site and the source of pathology (**Figure 1**).

Four converging lines of evidence that support this characterization are examined in turn below: the first-trimester origin of the structural lesion (Section 8.2.1); pre-clinical detectability of EOPE by placental biomarkers (Section 8.2.2); the causal sufficiency of placental factors alone to produce the phenotype (Section 8.2.3); and the therapeutic window that opens with placentation and closes once the placenta is structurally set (Section 8.2.4).

#### 8.2.1 First-trimester placentation defects

The original demonstration that EOPE placentas show defective spiral artery remodeling was made by Brosens, Robertson, and Dixon in 1972 (64). Subsequent quantitative work confirmed that trophoblast invasion depth in the placental bed is substantially reduced in EOPE compared to normotensive controls, and that the converted-vessel fraction is correspondingly diminished (65). The rheological consequences of incomplete spiral artery remodeling — pulsatile, high-resistance perfusion of the intervillous space generating shear-stress and oxidative injury to the syncytiotrophoblast — have been mathematically modeled by Burton and colleagues and shown to be sufficient to produce the chronic ischemia and ischemia–reperfusion injury that characterize the EOPE placenta (66). These findings establish that the EOPE-relevant lesion is anatomical and arises during placentation itself, not at the time of clinical presentation.

#### 8.2.2 Pre-clinical detectability

If EOPE were primarily a maternal cardiovascular disease, it should not be detectable by placental biomarkers before maternal symptoms appear. Yet first-trimester combined screening at 11–13 weeks — using maternal characteristics, mean arterial pressure, uterine artery pulsatility index, pregnancy-associated plasma protein-A (PAPP-A), and placental growth factor (PlGF) — detects approximately 96% of pregnancies that will develop preterm preeclampsia (<34 weeks), at a 10% false-positive rate (67). The detection rate for term preeclampsia using the same algorithm is markedly lower (~36%), revealing a striking asymmetry: EOPE leaves a placental-biomarker fingerprint at first-trimester screening that LOPE does not. Thus, the development of EOPE could be already in motion at 11–13 weeks — long before maternal blood pressure rises, proteinuria develops, and the PMIP. LOPE, by contrast, is largely undetectable at first-trimester screening, consistent with its developing in a placenta that is functionally normal until late gestation.

#### 8.2.3 Causal sufficiency: placental factor alone is sufficient

Three lines of evidence establish that placental factors alone are sufficient to produce the preeclampsia phenotype. First, complete hydatidiform mole — a placenta arising in the absence of a fetus — can produce a preeclampsia-like syndrome, demonstrating that the placental tissue is by itself sufficient to trigger the maternal syndrome. Second, removal of the placenta at delivery resolves preeclampsia within hours to days, identifying the placenta as the necessary source of the disease-driving factors. Third, and most directly relevant to the developmental origin of EOPE, transgenic mouse models expressing the human STOX1 transcription factor in the fetoplacental compartment alone — with wild-type maternal genotype — reproduce a severe preeclampsia phenotype with elevated soluble fms-like tyrosine kinase-1 (69). STOX1 was first identified through positional cloning of preeclampsia-affected Dutch families (68). The demonstration that fetoplacental expression alone is sufficient to cause the phenotype (69) is genetic-causal evidence for the placenta-as-disease-site interpretation.

#### 8.2.4 Therapeutic asymmetry

The pattern of which interventions work and which do not provides further evidence that EOPE is a placental disease whose therapeutic window closes once the placenta is structurally set. Aspirin initiated between 8 and 16 weeks of gestation, when trophoblast invasion is still ongoing, reduces the incidence of preterm preeclampsia by approximately 62% (ASPRE trial) (71); the same intervention initiated after 16 weeks shows weaker and inconsistent effect, and benefit is expected to be negligible when initiated after the PMIP, when the placental architecture is fixed — a pattern consistent with, though not uniquely explained by, the PMIP framework (71–73). Conversely, attempts to rescue an already-injured EOPE placenta in late gestation have systematically failed. The STRIDER trial of maternal sildenafil for severe early-onset fetal growth restriction was halted early for futility and a signal of neonatal pulmonary hypertension, with no improvement in perinatal outcomes (74). The asymmetry — EOPE prevention feasible only in early gestation, while EOPE rescue in established disease has failed — is consistent with the framework.

### 8.3 LOPE as host disease: post-PMIP metabolic mismatch

In contrast to the multi-strand evidence base for EOPE-as-organ-disease, the LOPE-as-host-disease argument follows a single mechanistic line of reasoning, developed below.

LOPE, in most cases, is proposed to occur in a placenta that has largely completed the Phase I→Phase II transition. The organ is frequently morphologically normal with properly-formed terminal villi and attenuated VSMs. Importantly, one cross-sectional molecular study (62) reported that UPR markers, AKT levels, and stress-pathway activation in LOPE placentas were indistinguishable from normotensive controls delivered at equivalent gestational ages — a finding that is consistent with the PMIP model but as yet has not been independently replicated, and which applies specifically to the molecular pathways examined in that study rather than to placental biology broadly. LOPE arises primarily from mismatch between the mature organ’s fixed functional capacity and excessive maternal cardiovascular or metabolic demand — driven by obesity, insulin resistance, chronic hypertension, advanced maternal age, or genetic predisposition. By ‘fixed functional capacity’ we mean the maximal achievable transport, hormone secretion, and metabolic flux — set by completed structural development at the PMIP (10, 75). Mechanistically, the LOPE bridge runs through reduced placental reserve as fetal demand peaks at ~35 weeks. Subclinical syncytiotrophoblast dysfunction in a structurally normal but maximally taxed organ produces a milder anti-angiogenic shift than EOPE, alongside maternal endothelial dysfunction that is partly intrinsic and unmasked by gestational hemodynamic and metabolic load. The maternal system is unable to accommodate the physiological demands of late pregnancy. Within this framework, LOPE is best conceptualized as a predominantly host-driven disease — where “host” denotes the maternal physiological system that hosts the placenta, not in the immunological sense of host–defense or graft-versus-host — in which the maternal cardiovascular/metabolic system is the primary site and source of pathology in most cases — while acknowledging that a subset of LOPE cases, particularly those with concurrent fetal growth restriction and abnormal angiogenic biomarkers, may represent delayed manifestation of placental-origin disease (see Section 9.6).

This framework must also account for the approximately 25% of LOPE cases that present with concurrent fetal growth restriction. In LOPE-FGR, the placenta itself is structurally and molecularly mature, and growth restriction reflects maternal supply limitation rather than placental architectural failure. Hemodynamic studies of preeclampsia with concurrent FGR have documented reduced maternal cardiac output, elevated systemic vascular resistance, and impaired uteroplacental perfusion in this subgroup (75, 79, 80). These features are consistent with a maternal cardiovascular delivery deficit to a competent placenta rather than an organ-intrinsic transport deficit. This distinction has direct management implications: maternal cardiovascular and metabolic optimization is the rational therapeutic target in LOPE-FGR, in contrast to the placentally-targeted interventions appropriate for EOPE-FGR.

Tong et al. have reviewed how impairments in STB differentiation, secretion, metabolism, and cellular heterogeneity contribute to preeclampsia (76). Within the PMIP framework, these dysfunctions take on temporal meaning. In EOPE, they reflect arrested Phase I fusogenic programming, whereas in LOPE they reflect failure of Phase II functional optimization, which depends on a fully matured STB layer. The comparative biological and clinical features of EOPE and LOPE within the PMIP framework are summarized in **Table 2**. The organ disease versus host disease distinction, while acknowledging phenotypic overlap, is not merely semantic. It has direct implications for how these conditions should be investigated, monitored, and ultimately treated, as discussed in Section 11.

**Table 2:** Comparative biological and clinical features of EOPE versus LOPE within the PMIP framework.

Domain	EOPE (<34 weeks)	LOPE (≥34 weeks)
Prevalence	~10–20% of PE cases	~80–95% of PE cases
Concurrent FGR rate	~60%	~25%
First-trimester spiral artery remodeling	Defective (maternal vascular malperfusion)	Largely normal
Villous morphology	Accelerated villous maturation/placental villous hypermaturation (PVH); syncytial knots often >30%	Morphologically normal for gestational age
Vasculosyncytial membrane	Variable; often compromised by surface-area deficit	Attenuated; functionally optimized
sFlt-1/PlGF rule-in cutoff	≥85	≥110
PlGF trajectory	Severely suppressed	Mildly suppressed to normal
hPL	Reduced relative to placental mass	Appropriate for mass
UPR markers (P-IRE1α, ATF6, XBP-1, GRP78, GRP94)	Markedly activated	Indistinguishable from normotensive controls (62)
MAPK stress markers (P-p38), HSP70	Activated	Indistinguishable from controls (62)
AKT (cell survival pathway)	Selectively suppressed (62)	Normal (62)
Telomere length (GA-adjusted)	Shortened in trophoblasts (61)	Near-normal (60, 61)
Senescence markers (P16, SA-β-Gal)	Markedly elevated; constitutive (ischemic-driven)	Mildly elevated; reactive (oxidative-stress-driven; rescuable by SOD (53))
Placental calcification	Premature Grade III before 36 weeks (pathological)	Physiological Grade II/III after 36 weeks (benign)
Maternal hemodynamics	Low cardiac output, high systemic vascular resistance, volume-depleted (75, 79, 80)	High cardiac output, normal/low SVR, volume-overloaded (75, 79, 80)
Dominant maternal risk factors	Prior PE, chronic hypertension, autoimmune disease, thrombophilia, primigravidity (10)	Obesity, gestational diabetes, advanced maternal age, chronic hypertension, multiparity (10)
Therapeutic target	Placenta (e.g., aspirin <16 weeks optimal; angiogenic rebalance)	Maternal cardiovascular and metabolic systems
PMIP-framework classification	Insufficient Phase I with reactive Phase II activation (organ disease)	Post-PMIP metabolic/CV mismatch (host disease)

FGR, fetal growth restriction; GA, gestational age; hPL, human placental lactogen; MAPK, mitogen-activated protein kinase; PE, preeclampsia; PMIP, Placental Maturation Inflection Point; PVH, placental villous hypermaturation; SOD, superoxide dismutase; STB, syncytiotrophoblast; SVR, systemic vascular resistance; UPR, unfolded protein response.

## 9 Counter-arguments and limitations

### 9.1 Circularity of the 34-week cutoff

A potential critique concerns circularity: the 34-week clinical cutoff was established through epidemiological validation (10), and subsequent biomarker thresholds (e.g., gestational-age-specific sFlt-1/PlGF cutoffs (50–52)) were calibrated around this boundary. One might argue that biological evidence clustering at 34 weeks merely reflects studies designed around a pre-existing clinical threshold. Two considerations weigh against this concern. First, the structural evidence drawn from placental morphometry and villous stereology (16, 17, 23, 25, 26, 28, 29) was developed independently of the preeclampsia literature, in the context of normal placental developmental biology. Second, and most importantly, the molecular stress pathway inflection reported by Yung et al. (62) — in which late-onset preeclampsia placentas are molecularly indistinguishable from normotensive controls in UPR, MAPK, and AKT activation, while early-onset preeclampsia placentas are profoundly divergent — constitutes evidence independent of any clinical threshold-setting. It should be acknowledged, however, that the study design of (62) was powered to detect differences at the 34-week threshold and could not have detected boundary inflections at nearby gestational ages; prospective continuous-gestational-age designs would resolve this limitation. The molecular discontinuity at 34 weeks was detected through direct biological comparison, not through threshold calibration. While the clinical cutoff may partially reflect epidemiological stratification, the 34–36 week biological transition has independent support from developmental morphometry and unbiased molecular comparison.

### 9.2 The 34-week cutoff as a spectrum, not a boundary

As summarized in **Table 3**, several studies have cautioned that EOPE and LOPE exist on a continuum rather than as discrete entities. A 2022 review explicitly stated that differences lie on a spectrum. The earlier preeclampsia develops, the more severe the angiogenic imbalance (77). There is no consistent international classification for the cutoff (78). Our framework proposes a biological inflection zone (approximately 34–36 weeks) within which the transition occurs. The clinical cutoff at 34 weeks approximates the onset of this transition, not its completion.

**Table 3:** Counter-arguments to the PMIP framework: evidence, responses, and research priorities.

#	Counter-argument	Evidence	PMIP response	Research needed
1	Circularity of the 34-wk cutoff	34-wk cutoff established epidemiologically (10); biomarkers calibrated around it (50–52); biology may be circular to threshold.	Pillar 7b (62) was detected by unbiased biological comparison; morphometry (16, 17, 23, 25, 26, 28, 29) derives from normal-pregnancy biology, not PE literature.	Unbiased prospective biomarker discovery using continuous gestational-age sampling without GA-conditioned stratification.
2	34-wk cutoff is a spectrum, not a boundary	AJOG 2022: “34 weeks is not a hard cutoff”; differences on a continuum (77); ISSHP 2021 allows both 34 and 37 wk classifications (78)	The PMIP is a biological transition zone (~34–36 wk), not a deterministic switch. Individual variation is expected.	Serial multi-modal studies defining PMIP timing across populations
3	FGR-based classification is better	PE with FGR has different hemodynamics at any GA (75, 79, 80); ~60% EOPE vs ~25% LOPE have concurrent FGR (4)	The frameworks compete on which axis is primary. FGR-stratified phenotypes are themselves explicable as Phase I arrest (with FGR) versus Phase II mismatch (without FGR).	Prospective comparison of FGR-based vs GA-based vs biomarker-based PE classification
4	MRI shows linear growth, not plateau	16/20 women showed continued linear growth to 38 wk in the most rigorous longitudinal study (18)	Phase II involves a qualitative shift (VSM optimization), not volumetric cessation. Seven-pillar convergence establishes the PMIP.	Larger longitudinal MRI (n>100) with concurrent biomarkers through 42 wk
5	Telomere length is GA-dependent, not PE-specific	Yang et al., 2022 (n=224): TL correlates with GA, not PE severity (p=0.20 after adjustment) (60)	TL attrition is programmed aging, consistent with PMIP. EOPE shows premature activation of this normal process.	Cell-type-specific longitudinal TL analysis within same pregnancies
6	Some LOPE cases have placental pathology	A subset of ≥34 wk PE shows severe FGR, abnormal Doppler, and markedly elevated sFlt-1/PlGF ratio (5, 81, 82)	These cases likely represent Phase I failure with delayed manifestation. Angiogenic subtyping may be more accurate than GA alone.	Prospective angiogenic PE subtyping performed alongside conventional GA classification
7	Transcriptomic heterogeneity within subtypes	Single-cell transcriptomic and spatial multi-omics studies reveal molecular subclasses crossing the 34-wk boundary (83)	The PMIP is a transition window; individual timing varies by genetics, metabolism, and other factors without invalidating the framework.	Single-cell/spatial transcriptomics spanning the 30–38 wk window specifically
8	Neonatal viability threshold as alternative explanation	Neonatal morbidity (RDS, IVH, NEC, BPD) decreases markedly after 34 wk; cutoff may reflect neonatal-risk gradient (70, 84).	Not mutually exclusive: clinical adoption may have been neonatal-driven, but Pillar 7b (62) was observed directly in placental tissue.	Prospective placental maturation measurements decoupled from delivery indication.
9	Confounding by delivery gestational age	EOPE placentas sampled at 28–33 wk vs LOPE at 34–41 wk; LOPE cannot be ethically sampled earlier — confounds all comparisons.	Yung (62) used preterm non-PE controls, separating disease-divergence from preterm placental biology.	Larger sample sizes with carefully matched controls across 30–38 wk; longitudinal imaging/biomarker designs that do not require tissue sampling.

### 9.3 The FGR-based alternative classification

Multiple groups have proposed that the real distinction between preeclampsia phenotypes is not gestational age at onset but the presence or absence of fetal growth restriction (75, 79, 80). In that framework, PE with FGR shows low cardiac output, high vascular resistance, and depleted intravascular fluid, whereas PE without FGR shows high cardiac output, normal or low vascular resistance, and intravascular fluid overload (75, 79, 80). This classification competes with the gestational-age-based PMIP framework on which axis is biologically primary. We do not claim the two frameworks are simply complementary; rather, the FGR-stratified hemodynamic phenotypes themselves are explicable as Phase I arrest (with FGR) versus Phase II demand–supply mismatch (without FGR). This generates a falsifiable prediction. Among PE cases without FGR, molecular and morphometric signatures should track gestational age (PMIP-consistent), whereas among PE cases with FGR, signatures should track Phase I arrest regardless of gestational age at delivery. The strong correlation between FGR and EOPE (~60% versus 25% in LOPE (4)) reflects that pre-PMIP placental dysfunction inherently limits fetal nutrient supply, making FGR an expected consequence.

### 9.4 Placental volume data: linear or plateauing?

Mathematically, the MRI volumetric data show deceleration (decreasing first derivative on cubic polynomial fit), not an asymptotic zero-slope plateau. Not all MRI volumetric studies demonstrate a clear volumetric plateau. In a longitudinal study, 16 of 20 women showed continued linear placental volume growth from week 14 through 38, with only 4 women showing plateau behavior (18). This raises the possibility that the Phase I→Phase II transition may not involve a cessation of volumetric growth per se, but rather a qualitative shift in what is being produced — from new functional parenchyma to structural optimization of existing tissue. The volumetric data alone are insufficient to define the PMIP. It is the convergence with endocrine (hPL near-plateau), molecular (stress pathway inflection), histological (VSM attenuation), and imaging (calcification threshold, T2* decline) data that makes the case. Larger prospective MRI volumetric studies with serial measurements extending through 40–42 weeks are needed to precisely characterize the volumetric trajectory.

### 9.5 Telomere length and preeclampsia

The relationship between placental telomere length and preeclampsia remains contested. Farladansky-Gershnabel et al. found shorter telomeres and increased senescence markers in EOPE versus LOPE placentas (61), whereas Yang et al. demonstrated in a large cohort (n=224 vs. n=26) that telomere length shortening is primarily associated with gestational age rather than preeclampsia status (60). Two mechanisms may reconcile this discrepancy. First, syncytiotrophoblast senescence — where short telomeres may reflect ischemic damage rather than replicative aging — is distinct from bulk placental telomere biology. The studies may have sampled these compartments differently. Second, the smaller cohort may have been underpowered to detect the gestational-age effect that dominates in the larger study, such that what appears as a disease-specific effect in (61) is partly attributable to maturation-associated telomere dynamics. The broader implication that cross-sectional placental studies face gestational-age confounding is addressed in Section 9.9. Resolution requires cell-type-specific telomere analyses in prospective cohorts with matched third-trimester controls.

### 9.6 Late-onset cases with placental pathology

An important complication is that some cases classified as LOPE (≥34 weeks) exhibit EOPE-like features — severe FGR, abnormal uterine artery Doppler, elevated sFlt-1/PlGF ratio (5, 81). These may represent true placental disease with delayed clinical manifestation rather than “maternal” preeclampsia. The placental pathology likely originated in Phase I but only met diagnostic criteria after 34 weeks. Their existence complicates the clean dichotomy proposed by the PMIP framework and suggests that a subset of LOPE cases may be biologically misclassified by gestational age alone. Reclassification based on angiogenic biomarker profiles may distinguish “angiogenic” PE (high sFlt-1/PlGF, placental origin) from “non-angiogenic” PE (normal ratio, maternal origin) (82), and more accurately capture the true biological phenotype and align with the PMIP framework. Building on this distinction, LOPE presenting with FGR may be stratified into placental-origin (late-presenting EOPE) and maternal-origin (supply-chain restriction of a normal placenta) subtypes based on molecular and morphometric criteria. Direct support for this stratification comes from a recent cohort analysis of women with chronic hypertension. Those with fetal growth restriction confirmed by abnormal Dopplers at 35–36 weeks — within the PMIP zone — progressed to superimposed preeclampsia significantly more often than propensity-matched controls, whereas small-for-gestational-age fetuses with normal Dopplers did not (93). This finding aligns with the prediction that placental vascular insufficiency persisting into the PMIP window identifies a placental-origin subgroup among late-onset cases.

### 9.7 Transcriptomic heterogeneity within PE subtypes

Recent single-cell transcriptomic and spatial multi-omics studies have revealed considerable molecular heterogeneity within both EOPE and LOPE that does not perfectly align with a strict 34-week cutoff (83, 92). Molecular subclasses of preeclampsia may be defined by specific pathway activation patterns, immune cell profiles, or metabolic signatures that cross the gestational age boundary. The PMIP framework should therefore be understood as identifying a biological window — a molecular transition zone — rather than a switch at a fixed gestational age. Individual variation in the timing of this transition likely produces a distribution of PMIP timing across the population. The 34-week clinical cutoff captures the central tendency of this distribution, not a fixed timepoint for every individual. Future single-cell and spatial transcriptomic studies spanning the 30–38 week window will be essential to characterize the molecular heterogeneity within the PMIP transition zone.

### 9.8 The neonatal viability threshold as an alternative explanation

A competing explanation is that the 34-week cutoff reflects discontinuity in neonatal outcomes rather than placental biology. Neonatal morbidity and mortality — including respiratory distress syndrome, intraventricular hemorrhage, necrotizing enterocolitis, and bronchopulmonary dysplasia (70) — decrease markedly after 34 weeks of gestation, and the cutoff may have been adopted clinically because expectant management decisions hinge on this neonatal risk gradient (84). Under this alternative, the differential profiles between EOPE and LOPE would reflect selection bias introduced by clinical management thresholds rather than discrete placental biology. We consider the two explanations not mutually exclusive. The clinical cutoff may have been adopted initially for neonatal-outcome reasons, while the underlying placental biology, documented here, provides independent grounding that renders the cutoff justified. Three lines of argument weigh against this counter-explanation. First, the molecular discontinuity demonstrated by Yung et al. (62) is a placental tissue phenomenon. UPR pathway activation, AKT suppression, and stress-protein expression are intrinsic to the delivered organ and cannot be inferred from neonatal outcomes. Second, the molecular pattern in ≥34-week PE placentas is indistinguishable from normotensive controls, which is the inverse of what neonatal-risk arguments would predict. If neonatal viability gradients drove the 34-week boundary, the placental molecular state would not also recapitulate this boundary. Third, several non-neonatal evidence pillars (structural stereology, endocrine plateau, calcification trajectory) are set *in utero* before delivery is contemplated and cannot be downstream of neonatal-outcome decision making. While we acknowledge that clinical adoption of the 34-week threshold may have been driven historically by neonatal considerations, the convergence of placental biological evidence at the same boundary is independent of the neonatal-risk gradient.

### 9.9 Confounding by delivery gestational age

Cross-sectional placental studies face an inherent confound: EOPE tissue is collected at earlier gestational ages (≤33 weeks) than LOPE tissue (34–41 weeks), because LOPE placentas cannot ethically be sampled before 34 weeks. Any EOPE–LOPE differential could therefore reflect normal gestational-age biology rather than disease-specific pathology. This confound is not unique to the PMIP framework. It affects every comparative study in the placental preeclampsia literature. The telomere discussion in Section 9.5 illustrates one consequence, in which disease-specific and maturation-associated effects have proved difficult to separate (60, 61). Two methodological strategies partially address the confound. The first, exemplified by the telomere studies, uses gestational-age-matched controls for both EOPE and LOPE placentas. The second, exemplified by Yung et al. (62), incorporates preterm non-preeclamptic controls so that the observed molecular divergence of EOPE placentas is distinguishable from normal preterm placental biology. The parallel finding that LOPE placentas are indistinguishable from term normotensive controls in UPR, MAPK, and AKT activation is not readily attributable to gestational-age confounding in the opposite direction. However, neither strategy eliminates the confound entirely. Replication with larger samples across the 30–38 week window, ideally including longitudinal imaging and biomarker data that do not require tissue sampling, is a key research priority.

### 9.10 Overall limitations

The PMIP is presented as an integrative hypothesis based on convergent evidence, and several caveats must be acknowledged. The precise timing varies between studies (32–36 weeks); whether this reflects methodological differences, biological heterogeneity, or both, remains unclear. Larger prospective cohorts with cell-type-specific analyses are needed. Finally, the integration of seven evidence domains is performed qualitatively; future quantitative integration (e.g., Bayesian meta-analytic models incorporating all data streams) would strengthen the case but is not feasible given the heterogeneity of measurement approaches across domains.

## 10 Future research directions

The PMIP framework generates testable hypotheses that require prospective investigation. These fall into two categories: methodological research priorities (i.e., the types of studies needed to characterize the transition at higher resolution) and specific falsifiable predictions the framework makes. The testing of these hypotheses could validate or refute it as a biological framework.

### 10.1 Methodological research priorities

Six lines of investigation would strengthen the framework. First, no single study has prospectively measured placental volumetric growth, villous morphometry, molecular differentiation markers (p45 NF-E2, GCM1), and endocrine output (hPL, PlGF) in the same cohort across gestation. To confirm temporal convergence at the PMIP, a longitudinal multi-modal assessment study — combining MRI volumetry, serum biomarkers, and stereological and molecular analysis — spanning weeks 24–40 in both normal and high-risk pregnancies would be definitive. Such a study would require collaboration among maternal-fetal medicine specialists, placental pathologists, and imaging specialists to provide a comprehensive temporal map of the PMIP transition among individuals.

Second, single-cell and spatial transcriptomic studies of the placenta across the 30–38 week window could identify molecular triggers of the Phase I→Phase II transition and the signaling pathways coordinating volumetric plateau, VSM attenuation, and endocrine shift. Such studies would clarify whether the PMIP is regulated by a single master transcriptomic event or by the convergence of multiple molecular events.

Third, whether maternal constitutional factors (e.g., obesity, hypertension, diabetes, altitude of residence) alter the timing of the PMIP deserves systematic investigation. This may explain much of the heterogeneity observed in LOPE presentation and inform personalized risk stratification.

Fourth, whether placental-targeted agents administered before versus after the PMIP have differential efficacy is a critical translational question, particularly as novel sFlt-1/PlGF and other placental pathway therapeutics enter clinical development. The ASPRE trial and subsequent meta-analyses have shown that aspirin reduces preterm PE but not term PE (71–73). That is consistent with the PMIP-dependent therapeutic efficacy view and provides proof of principle for this line of analysis.

Fifth, advanced MRI techniques, including blood-oxygen-level-dependent (BOLD) MRI, IVIM (intravoxel incoherent motion) diffusion imaging, and T2* relaxometry, should be applied longitudinally across the third trimester to identify imaging signatures of the Phase I→Phase II transition. The results could eventually enable real-time, non-invasive assessment of placental maturation status.

Sixth, prospective validation of whether PMIP timing varies by ethnicity, fetal sex, parity, or environmental factors (altitude, pollution exposure) could refine the framework and improve personalized risk assessment for preeclampsia subtypes.

### 10.2 Falsifiable predictions

Beyond methodological priorities, the PMIP framework makes four falsifiable predictions testable with available technology. Demonstration would validate the framework; failure would argue for revision or rejection.

First, prospective multi-domain studies should show that hPL near-plateau, PlGF decline, volumetric deceleration, and calcification acceleration occur within the same 2–3 week window in individual women, not merely at similar gestational ages averaged across populations. Demonstration of within-individual temporal convergence would substantially strengthen the case that these markers reflect a single underlying developmental transition rather than coincidentally overlapping trajectories.

Second, metabolic dysregulation is a well-established risk factor for LOPE but shows weak or absent association with EOPE (10). This suggests that metabolic state may modulate placental maturation timing itself. Maternal hyperinsulinemia and altered insulin-like growth factor (IGF) signaling are known to promote placental overgrowth (placentomegaly), hypervascularization, and altered trophoblast proliferation (85–88). Within the PMIP framework, such metabolic states may artificially prolong Phase I parenchymal expansion and delay the normal Phase I→Phase II transition or may overwhelm the fixed functional capacity of a normally matured Phase II placenta with excessive metabolic demand. Either mechanism would explain why LOPE in metabolically compromised mothers presents with different severity, biomarker profiles, and outcomes than LOPE in metabolically healthy mothers, and why BMI shows a stronger association with LOPE than with EOPE (10). This can be tested through serial MRI volumetry plus metabolic biomarker profiling in obese versus normal-weight cohorts. Demonstration of delayed or attenuated PMIP transitions in the metabolically compromised arm would validate this component. Failure to detect such differences would argue against metabolic modulation.

Third, if the PMIP represents a genuine biological transition, EOPE placentas delivered before 34 weeks should show morphometric and molecular profiles of incomplete Phase I — limited terminal villus formation, residual cytotrophoblast proliferation, incomplete VSM attenuation. On the other hand, LOPE placentas delivered after 36 weeks should show completed Phase II signatures. This is testable against existing placental biobanks with gestational-age metadata.

Fourth, identification of molecular or endocrine triggers driving the Phase I→Phase II transition at 34–36 weeks constitutes a high-priority falsifiable prediction. Candidate mechanisms worth prospective investigation include peak fetal weight velocity at approximately 33–35 weeks (24), which may exhaust placental reserve capacity as demand outpaces supply; and progressive trophoblast telomere attrition (60, 61) that may reach a threshold for senescence activation. We note that the fetal cortisol trajectory, although a plausible candidate based on its well-documented maturational roles in lung, gut, and neuroendocrine systems, shows its steep rise after 36 weeks (from ~20 ng/mL at 35–36 weeks to ~45 ng/mL at term (89)) rather than across the 34–36 week PMIP window. This timing suggests that cortisol is more likely a downstream post-PMIP fetal maturation signal. Identification of the proximate trigger would transform the PMIP from a descriptive framework into a mechanistically anchored developmental event, and this could enable targeted intervention strategies not currently available.

## 11 Translation to clinical practice: a multi-modality assay panel for PMIP-stratified diagnosis

The PMIP framework is a biological synthesis, but a recurring question from practicing physicians is whether it can be integrated into an assay panel for diagnosis and treatment stratification. We address this question because clinical-translation logic flows directly from the seven-pillar architecture. Each pillar represents a measurable biological correlate of either Phase I integrity or the Phase I→II transition, and each measurement is most informative within a specific gestational age window. We do not propose a clinical guideline; ACOG, ISSHP, and FIGO guidelines remain authoritative. Rather, we sketch the research-and-translation panel the framework generates, distinguishing clinical-tier (regulatory-cleared, in routine use) from research-tier (validated in research settings but not yet implemented at scale) assays.

Pre-PMIP screening (16–30 weeks) — predicting EOPE risk. Clinical-tier assays here are well established. The first-trimester combined screen, validated in the ASPRE trial, integrates maternal characteristics, mean arterial pressure, uterine artery pulsatility index, and PlGF (71–73). The sFlt-1/PlGF ratio at 24–34 weeks, with the PROGNOSIS rule-out cutoff of ≤38 (negative predictive value 98.6% for short-term preeclampsia within one week (51); 99.3% in the original multinational PROGNOSIS cohort (96)), is also clinical-tier and CE-marked in Europe (51). Research-tier assays for this window include mid-trimester placental volume by 3D ultrasound or MRI (18, 19), first-trimester power-Doppler characterization of functional spiral artery count and jet dispersion index (the latter discriminating pregnancies that subsequently develop PE and FGR as early as 11–14 weeks (94)), and emerging spatial-transcriptomic signatures of impaired spiral artery remodeling (92). Failure of Phase I parenchymal expansion is the biological rationale for why all of these screen positively for EOPE specifically rather than for preeclampsia in general.

Trans-PMIP monitoring (30–36 weeks) — mechanism-based stratification of emerging preeclampsia. The framework offers greatest interpretive leverage in this window, because biomarker outputs of a maturing placenta differ qualitatively from those of an arrested one. Within the clinical tier, sFlt-1/PlGF dynamics are most informative when measured serially. A ratio that fails to rise into the expected post-PMIP range while clinical features develop suggests Phase I arrest, whereas a sharply rising ratio above the higher rule-in cutoff (often ≥85) in a previously normotensive woman is consistent with the post-PMIP physiological transition (50–52). hPL trajectory monitoring (Pillar 4) is conceptually attractive — failure to reach near-plateau by 34 weeks would directly indicate Phase I arrest — but remains research-tier. Longitudinal hPL reference curves with sufficient gestational resolution are not yet standardized (45, 46). Research-tier assays for this window also include placental T2*-MRI for oxygenation and Grannum grading with the explicit caveats noted in Section 6.1. Emerging serum biomarkers that discriminate onset subtypes by magnitude — such as SPARCL-1, whose levels are significantly lower in EOPE than in LOPE, consistent with more severe endothelial disruption in pre-PMIP placental disease (95) — may complement established angiogenic ratios in this window, although validation in larger longitudinal cohorts is needed.

Post-PMIP triage (≥34 weeks) — distinguishing EOPE-late-presentation from true LOPE. A clinically important question the framework helps formalize is whether a woman presenting after 34 weeks with new-onset hypertension and proteinuria has true LOPE (Phase II demand–supply mismatch in a structurally normal placenta) or delayed-presentation EOPE (delayed presentation of arrested-Phase I disease, unmasked as maternal compensation fails). The sFlt-1/PlGF ratio is again the only clinical-tier discriminator. Very high values, particularly in the presence of fetal growth restriction, are more consistent with arrested Phase I regardless of presentation timing. Research-tier confirmation requires post-delivery placental analysis of stereological VSM density (Pillar 2), molecular stress markers (Pillar 7b — UPR, MAPK, AKT activation), and senescence markers (Pillar 7a) to collectively profile the placenta status. These are not bedside assays, but they are increasingly accessible in academic centers and represent the natural endpoint for phenotype-confirmation studies.

Therapeutic stratification follows from diagnostic stratification, and EOPE-suspected phenotypes call for placentally-targeted interventions within the responsive window. Low-dose aspirin remains the only clinical-tier option, and recent debate over delivery timing (90, 91) aligns with the framework’s prediction that cost-benefit balance differs across the PMIP boundary. LOPE-suspected phenotypes call for maternal cardiovascular and metabolic optimization rather than placental intervention. The placenta in late LOPE is biologically intact, and treatment efforts targeting it are unlikely to add benefit. The framework also predicts that interventions trialed in mixed-phenotype cohorts may obscure true effects. A drug active against arrested-Phase I biology will appear ineffective if tested across a predominantly LOPE population, and vice versa. Trial enrichment by PMIP phenotype — using the assay panel above — is therefore a research priority that the framework directly motivates.

However, two caveats bound this panel. First, the groupings reflect the current state of biomarker development; many research-tier components require prospective validation, reference-range standardization across diverse populations, and demonstration of decision-impact before clinical translation. Second, the panel is gestational-age-anchored, which means that women presenting near the 34–36 week boundary occupy an interpretive grey zone — exactly the population in whom the PMIP framework predicts maximum biological heterogeneity. Honest acknowledgment of this grey zone is essential. The framework provides interpretive structure for this transition, not a deterministic rule. **Table 4** summarizes the panel organized by gestational age window, pillar, assay tier, and intended decision support.

**Table 4:** Multi-modality assay panel for PMIP-stratified diagnosis.

GA window	Pillar	Assay	Tier	Decision support
Pre-PMIP (16–30 wk)	Pillar 1	Mid-trimester placental volume (3D US/MRI)	Research	Detects impaired Phase I parenchymal expansion
Pre-PMIP (16–30 wk)	Pillar 5	First-trimester combined screen (PlGF + UtA-PI + MAP)	Clinical	Identifies high-risk women for aspirin prophylaxis
Pre-PMIP (16–30 wk)	Pillar 5	sFlt-1/PlGF ratio (PROGNOSIS ≤38 rule-out, 24–34 wk)	Clinical	Rule-out short-term PE (NPV 98.6%)
Pre-PMIP (16–30 wk)	Pillar 3	Spatial-transcriptomic placental signatures	Research	Phase I integrity at maternal–fetal interface
Trans-PMIP (30–36 wk)	Pillar 5	Serial sFlt-1/PlGF dynamics	Clinical	Distinguishes Phase I arrest vs Phase II transition
Trans-PMIP (30–36 wk)	Pillar 4	Longitudinal hPL trajectory	Research	Failure to reach near-plateau indexes Phase I arrest
Trans-PMIP (30–36 wk)	Pillar 1	Placental T2*-MRI (oxygenation)	Research	Detects placental hypoxia/ischemia
Trans-PMIP (30–36 wk)	Pillar 6	Grannum grading by ultrasound	Research	Premature calcification flag (with caveats; Section 6.1)
Post-PMIP (≥34 wk)	Pillar 5	sFlt-1/PlGF ratio with FGR status	Clinical	Very high ratio + FGR favors EOPE-late-presentation
Post-PMIP (≥34 wk)	Pillar 2	Stereological VSM density (delivered placenta)	Research	Direct organ phenotyping post-delivery
Post-PMIP (≥34 wk)	Pillar 7b	UPR/MAPK/AKT markers (delivered placenta)	Research	Confirms Phase I arrest molecular signature
Post-PMIP (≥34 wk)	Pillar 7a	Senescence markers (telomere, p16, SA-β-gal)	Research	Distinguishes premature vs scheduled senescence
Any GA	Integration	PMIP phenotype assignment for trial enrichment	Research	Avoids mixed-cohort dilution of intervention effects

3D US, three-dimensional ultrasound; FGR, fetal growth restriction; GA, gestational age; MAP, mean arterial pressure; MRI, magnetic resonance imaging; NPV, negative predictive value; PE, preeclampsia; PlGF, placental growth factor; PMIP, Placental Maturation Inflection Point; SA-β-gal, senescence-associated β-galactosidase; sFlt-1, soluble fms-like tyrosine kinase-1; UPR, unfolded protein response; UtA-PI, uterine artery pulsatility index; VSM, vasculosyncytial membrane.

Assays are organized by gestational age window and pillar, with tier (Clinical = regulatory-cleared and in routine use; Research = validated in research settings but not yet implemented at scale) and intended decision support.

## 12 Implications for therapeutic windows, biomarkers, and trial design

Even prior to prospective validation, the PMIP framework suggests four potential translational directions, as it proposes a developmental-biologic interpretation for patterns that current obstetric practice manages empirically. Section 11 addressed diagnostic stratification; this section turns to the therapeutic and trial-design implications that follow from the same PMIP logic. These directions are presented as research hypotheses and conceptual scaffolding rather than as clinical recommendations.

First, it provides a biological rationale for gestational-age-specific biomarker cutoffs already in clinical use. The sFlt-1/PlGF ratio cutoffs differ before and after 34 weeks (50–52). The PMIP framework explains why: the same biomarker reports on two distinct biological processes depending on when it is measured. In arrested Phase I (EOPE), elevated sFlt-1 reflects maladaptive anti-angiogenic signaling from a placenta whose angiogenic program was never completed. After the PMIP (LOPE), elevated sFlt-1 reflects senescence-driven release from a structurally mature organ — a physiological rather than developmental signal. If the PMIP framework is validated prospectively, clinicians might consider not only whether the ratio is above or below a threshold but whether the placenta is likely pre- or post-PMIP, as the biological significance of the same ratio value may differ across these windows. This also has implications for trial design. Aggregating biomarker-positive women across gestational ages may obscure heterogeneous responses to placentally-targeted versus maternally-targeted interventions.

Second, the PMIP framework informs therapeutic-window optimization. Placental-dysfunction interventions are most effective when administered before the PMIP, while the organ is still growing its vascular supply. Aspirin illustrates this principle (as detailed in Section 8.2.4): initiated between 8 and 16 weeks, it reduces preterm preeclampsia by ~62% (ASPRE trial), while initiation after 16 weeks produces weaker inconsistent benefit (71–73). We therefore propose, as a research hypothesis, that the 8–16 week window may represent the primary intervention window for placentally-targeted therapies — aligned with active trophoblast invasion and spiral-artery remodeling (**Figure 1**) — with a secondary 16–30 week window for angiogenic-support interventions aimed at sustaining Phase I parenchymal expansion. Conversely, LOPE interventions should target the maternal cardiovascular/metabolic system rather than the placenta, which is functionally normal in most LOPE cases.

Third, the PMIP framework would reframe surveillance. Serial PlGF monitoring may be most informative during Phase I, when the angiogenic program is active and PlGF suppression is a sensitive indicator of placental dysfunction. After the PMIP, the declining PlGF is physiological, and the late-pregnancy assessment may require biomarkers that reflect placental functional capacity or maternal cardiovascular adaptation.

Fourth, the PMIP framework provides a conceptual tool for distinguishing EOPE versus LOPE management priorities. In EOPE, the central question is whether continued *in-utero* stay benefits the fetus given the high risks of extreme prematurity and the limited regenerative potential of an arrested placenta. For LOPE, the central question is whether continued gestation is tolerable for the maternal cardiovascular system. The framework does not change current management algorithms governed by ACOG, ISSHP, and FIGO guidelines, but it offers a biological narrative that may help clinicians explain to patients why early-onset and late-onset disease are managed differently. Recent debate over delivery timing in preeclampsia (90, 91) is consistent with this framing. Planned-delivery decisions before and after the PMIP zone reflect different biological realities — incomplete versus completed placental maturation.

## 13 Conclusions

The 34-week cutoff that distinguishes early-onset from late-onset preeclampsia may reflect an inflection in placental developmental biology — the Phase I (parenchymal expansion) to Phase II (functional optimization) transition. Seven lines of evidence of varying strength converge on a developmental transition zone at 34–36 weeks. Within this framework, EOPE is consistent with an arrested Phase I pattern in which defective spiral artery remodeling prevents the placenta from completing structural maturation and generates downstream disease signaling. LOPE, in most cases, is consistent with a post-PMIP mismatch pattern in which a largely mature placenta’s functional capacity is exceeded by maternal cardiovascular and metabolic demand; mixed and transitional phenotypes are acknowledged. The framework generates testable hypotheses with potential future implications for biomarker interpretation, therapeutic window selection, and clinical trial design. The convergence of multiple evidence streams provides a substantive conceptual foundation, but prospective longitudinal multi-modal studies are required to validate or refute the framework. We hope it serves as a useful conceptual tool for clinicians and investigators working to improve outcomes for women with preeclampsia worldwide.

## Data Availability

No original data were generated in this study. All data discussed are derived from previously published studies cited in the reference list.

## Glossary

3D: three-dimensional
ACOG: American College of Obstetricians and Gynecologists
AKT: protein kinase B
AMPKα: AMP-activated protein kinase alpha
aOR: adjusted odds ratio
ASPRE: Combined Multimarker Screening and Randomized Patient Treatment with Aspirin for Evidence-Based Preeclampsia Prevention
ATF6: activating transcription factor 6
BMI: body mass index
BOLD: blood-oxygen-level-dependent
BP: blood pressure
CE: Conformité Européenne
DVM: delayed villous maturation
EOPE: early-onset preeclampsia
EVT: extravillous trophoblast
FGR: fetal growth restriction
FIGO: International Federation of Gynecology and Obstetrics
GA: gestational age
GCM1: glial cells missing-1
GRP78: glucose-regulated protein 78
GRP94: glucose-regulated protein 94
HBC: Hamamatsu Birth Cohort
hCG: human chorionic gonadotropin
HERV: human endogenous retrovirus
hPL: human placental lactogen
HSP70: heat shock protein 70
IGF: insulin-like growth factor
IRE1α: inositol-requiring enzyme 1 alpha
ISSHP: International Society for the Study of Hypertension in Pregnancy
IUGR: intrauterine growth restriction
IVIM: intravoxel incoherent motion
LOPE: late-onset preeclampsia
MAPK: mitogen-activated protein kinase
MRI: magnetic resonance imaging
NF-E2: nuclear factor erythroid derived 2
NICE: National Institute for Health and Care Excellence
PE: preeclampsia
PlGF: placental growth factor
PMIP: Placental Maturation Inflection Point
PPC: premature placental calcification
PVH: placental villous hypermaturation
RR: relative risk
SA-β-Gal: senescence-associated beta-galactosidase
sFlt-1: soluble fms-like tyrosine kinase-1
SGA: small for gestational age
SK: syncytial knot
SMFM: Society for Maternal-Fetal Medicine
STB: syncytiotrophoblast
TL: telomere length
UPR: unfolded protein response
VEGF: vascular endothelial growth factor
VSM: vasculosyncytial membrane
XBP-1: X-box binding protein 1
